# Accuracy of a Novel Smartphone-Based Log Measurement App in the Prototyping Phase

**DOI:** 10.3390/s25185847

**Published:** 2025-09-19

**Authors:** Mirella Elias, Gabriel Osei Forkuo, Gianni Picchi, Carla Nati, Stelian Alexandru Borz

**Affiliations:** 1 Department of Forest Engineering, Forest Management Planning and Terrestrial Measurements, Faculty of Silviculture and Forest Engineering, Transilvania University of Brasov, Şirul Beethoven 1, 500123 Brasov, Romania; mirella.elias@unitbv.ro (M.E.); gabriel.forkuo@unitbv.ro (G.O.F.); 2 Institute of Bioeconomy, National Research Council, Via Madonna del Piano 10, 50019 Sesto Fiorentino, Italy; gianni.picchi@cnr.it (G.P.); carla.nati@cnr.it (C.N.)

**Keywords:** Tree Scanner, LiDAR sensor, log, volumes, digital measurements, manual measurement, traceability

## Abstract

Recently, the development of smartphone apps has resulted in a wide range of services being offered related to wood supply chain management, supporting decision-making and narrowing the digital divide in this business. This study examined the performance of Tree Scanner (TS)—a LiDAR-based smartphone app prototype integrating advanced algorithms—in estimating and providing instant data on log volume through direct digital measurement. Digital log measurements were conducted by two researchers, who each performed two repetitions; in addition to accuracy, measurement-time efficiency was also considered in this study. The results indicate strong agreement between the standard (manual) and digital measurement estimates, with an R^2^ > 0.98 and a low RMSE (0.0668 m^3^), as well as intra- and inter-user consistency. Moreover, the app showed significant potential for productivity improvement (38%), with digital measurements taking a median time of 21 s per log compared to 29 s per log with manual measurements. Its ease of use and integration of several key functionalities—such as Bluetooth transfer, remote server services, automatic species identification, the provision of instant volume estimates, compatibility with RFID tags and wood anatomy checking devices, and the ability to document the geographic location of measurements—make the Tree Scanner app a useful tool for integration into wood traceability systems.

## 1. Introduction

The forestry sector, like the primary sector, is facing novel and demanding challenges, including the climate crisis, an aging workforce, and globalization. Additionally, other factors hinder forest management, such as property fragmentation and technological development. The rapid advancement of information and communication technologies (ICTs) has fundamentally changed people’s knowledge and information behaviors. These changes stem from an unprecedented level of connectivity that characterizes people’s information environments [1].

Technological development presents both obstacles and opportunities for addressing these challenges. The transformation of a highly traditional context, such as the forestry sector, through the introduction of digital systems can lead to undesired consequences [2]. Typical barriers to the adoption of ICT solutions in rural areas include a lack of connectivity, as well as users’ and stakeholders’ fear and suspicion toward technology [3]. On the one hand, establishing stable connections has never been easier than it is today [4]; on the other hand, the aging human capital in rural communities may hinder their ability to embrace the benefits of ICT, unlike younger individuals, who are drawn to robust digitalization [5]. This disparity contributes to the narrowing of the digital divide [6].

In recent years, the rapid spread of mobile phones has provided opportunities to reach farmers, who are often remote, dispersed, and poorly serviced, by overcoming barriers of space and social standing. Mobile phones offer a range of services related to banking, payments, and commerce, as well as the ability to gather information in the form of images, audio recordings, graphics, videos, text, and functions such as marketplaces and social networking platforms [7]. In the agroforestry sector, the number of decision support tools available to farmers as smartphone apps has been steadily increasing. For instance, in India, 25 apps were used for various farming-related sectors, including horticulture, livestock management, farm operations, irrigation monitoring, soil health assessment, and agricultural marketing [8]. These tools range from equipment optimization (e.g., manufacturer-to-consumer instructions) to RFID management of livestock [9] and from global dairy herd management [10,11] to improvements in sustainability in agriculture and agronomic decision-making [12].

Equipped with high-quality RGB cameras, depth sensors, and LiDAR scanners, smartphones are becoming practical tools for close-range photogrammetry and remote sensing. Mobile devices have been effectively used to measure attributes such as diameter at breast height (DBH), tree height, basal area (BA), volume, and tree position. They utilize both passive and active sensing techniques, thus having the potential to improve the accuracy and efficiency of forest inventories. Tree attributes can be estimated either through indirect measurements via the post-processing of datasets or by direct methods using apps for mobile devices [13].

Significant advancements in forestry applications have included the use of general-purpose or dedicated apps for measuring and comparing the main biometrics of trees and logs with those obtained through manual methods [14,15,16]. While these apps are useful, they have several important shortcomings. They do not provide instant estimations of volume in the field, lack integration with other technologies such as RFID (radio frequency identification) tags, and miss other essential functionalities that allow for the sourcing, automatic data storage, and documentation of the geographic location necessary for traceability in modern management systems [17,18]. For effective digitalization in forestry, several key requirements must be met by mobile apps. These include performing accurately; being time-efficient; having the capability to store, process, and transfer digital data in real time; and, most importantly, integrating with other wood traceability technologies [19].

Considering these requirements, the Sintetic project [20] set the ambition to develop the Tree Scanner app as a comprehensive tool for single-item identification, sourcing, measuring, and traceability. In addition to estimating the volume of logs using a smartphone-embedded LiDAR sensor (real-time point cloud scanning) and identifying log species while documenting geographic location, the app is equipped with other essential functionalities. These include Bluetooth connectivity with the HITMAN acoustic tool for detecting wood stiffness and RFID (radio frequency identification) tag readers that uniquely identify each piece of wood. It is necessary to highlight that testing an app during the prototyping phase is an essential step in product development, as it is important for assessing functionality, accuracy, and practical use [21].

The aim of this study was to evaluate the performance of the Tree Scanner app (TS) as a digital measurement tool during its prototyping phase for log volume estimation by (i) assessing its accuracy compared to manual measurements, (ii) evaluating the internal consistency of the app’s estimates by considering two data sampling densities (0.1 and 0.5 m), (iii) assessing the agreement and intra-rater reliability of log volume estimates by repeating measurements by the same user, (iv) assessing the agreement and inter-rater reliability of log volume estimates by having two different users repeat the measurements, and (v) comparing the time efficiency of the app with that of manual log measurements.

## 2. Materials and Methods

### 2.1. App Description and Data Used in This Study

Tree Scanner (hereafter referred to as TS) is a smartphone-based application currently under development as part of the Sintetic (Single Item Identification for Forest Production, Protection, and Management) project [20]. The goal of this project is to harness the potential of digital technologies in supply chains that are still largely reliant on manual methods. To enhance its effectiveness, TS is being developed specifically for iPhone platforms, which incorporate a vertical-cavity short-range LiDAR sensor [22].

In summary, TS is designed to integrate various workflows that support critical functions within the supply chain, such as wood sourcing and traceability. The app enables users to measure the biometrics of trees and logs, process and save results on local devices, and transfer and store geo-referenced data in dedicated databases using state-of-the-art remote server connection technologies. For log measurement, it allows users to create measurement instances, take reference diameters at the ends of logs using deep learning models to recognize their profiles, and scan the logs using dedicated sensors.

The version utilized in this study was released in July 2024 and was tested via TestFlight (version 3.51, https://testflight.apple.com/join/IdnPkbIo, accessed on 15 September 2025), a tool developed by Apple (Cupertino, CA, USA) to facilitate the testing of app beta versions. In addition to estimating timber volume by applying intelligent sensors and algorithms to recognize features such as diameter, length, and tree species, the app is equipped with other essential functionalities. These include Bluetooth connectivity with the HITMAN acoustic tool for detecting wood stiffness and an RFID (radio frequency identification) tag reader to uniquely identify every tree or piece of wood. The TS app (version 0.7, Umeå, Sweden) allows users to collect, transfer, and store important sourcing, traceability, and quality information related to single tree and wood pieces.

Volume estimates were derived from the implementation of a Random Sampling and Consensus (RANSAC) algorithm [23], which operates on data collected in the form of LiDAR point clouds. The version tested in this study included two data-processing workflows, each producing a separate volume estimate. For each scanned log, the app automatically produced two datasets: one using measurements every 0.5 m along the log (hereafter called the first workflow), and the other using measurements every 0.1 m (hereafter called the second workflow). These outputs are hereafter referred to as sampling densities, which produced two separate volume estimates from the same scan, allowing us to assess the effect of different sampling densities on measurement reliability. Sampled diameters, log lengths, species, geographic locations, and timestamps were stored as .json files.

This study was designed to exert control over all factors that could potentially influence or compromise the test results. To mitigate any bias that might arise from processing data on the dedicated server, which could affect the measurement time estimates, in this study, the data was processed and stored directly on the mobile devices used for measurements, specifically, two iPhone 13 Pro Max smartphones having installed in advance the TestFlight and TS apps (Version 0.7). The data, saved as .json files, were later extracted and stored in a Microsoft Excel worksheet.

### 2.2. Location of the Study

**Figure 1:**
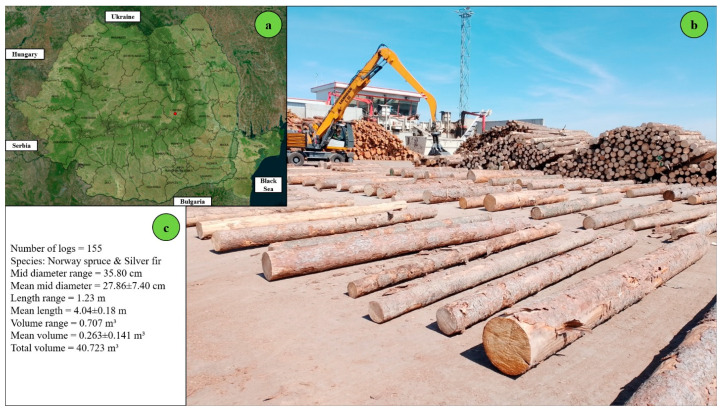
Location of the study area and characteristics of the sample used in this study: (**a**) national-level map showing the study site (red dot), (**b**) sample logs used for measurement (**c**) summary statistics of the sample, with volume estimates based on Huber’s formula.

The site setting and the arrangement of logs were chosen to limit the background noise as much as possible, because the presence of understory or grass would create artifacts in the collected point clouds or obstructions on the ground during measurement, which, in turn, could affect the computational workflow of the app and the time-consumption measurements. The species group was controlled as well by including in this study only coniferous logs to limit the number of variables. In forest operations, comparative time studies require control over factors such as the ground slope, ground condition, and piece size to be able to detect at a high degree of certainty the differences in estimates caused only by the technology used. Accordingly, the location of study was selected to accommodate all these requirements, except for log size, because a degree of variability in this feature was required to accommodate the reliability component of this study. This study was conducted in the log yard of an important wood processing company based in Romania, which is designated by the red dot in Figure 1a. The company handles material sourced both from domestic and foreign forests and typically works with short logs in the range of 3 to 4 m in length. A batch of logs representing a part of the sample used to take the measurements is illustrated in Figure 1b, while descriptive statistics of the sample, including volume estimates based on Huber’s formula, are provided in Figure 1c.

The sample under study consisted of 155 logs, which totaled an estimated volume of about 41 m^3^ and exhibited a wide variability in size. The logs were placed on the ground approximately 1 m apart from each other (Figure 1b). The ground was flat, paved with concrete, and cleaned before the experiment. The diameters measured at the midpoint ranged from 13 to 49 cm, with an average of 28 cm, while the mean length was close to 4 m. For detailed statistics concerning the biometric characteristics of the logs, please refer to Table S1 and Figure S1 (Supplementary Materials). The sky was mostly clear during the field data collection.

### 2.3. Experimental Design and Data Collection

**Figure 2:**
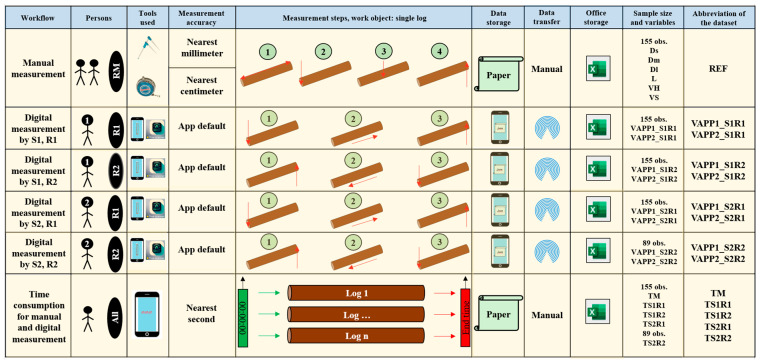
Description of the testing workflows implemented in the field.

The design of this study was primarily comparative, and the data were collected using both manual and digital measurements. Two researchers, hereafter referred to as Subject 1 (S1) and Subject 2 (S2), measured the logs as described in Figure 2. Both subjects conducted the measurement tasks in two replications. The order of log measurement remained consistent, irrespective of the subject or replication. In the first replication (R1), the logs were approached from one end, while in the second replication (R2), the logs were approached from the opposite end. S1 took measurements of all 155 logs in both replications, while S2 measured all logs in R1 and the last 89 logs in R2 due to space and production flow constraints in the factory.

For manual measurements (Figure 2), variables included the diameter at the small end (Ds), diameter at the middle (Dm), diameter at the large end (Dl), and log length (L). Volume estimates were calculated using Huber’s (VH) and Smalian’s (VS) formulae. Digital measurements were collected through the app with two sampling densities (VAPP1—digital volume estimate based on a sampling frequency of 0.5 m, VAPP2—digital volume estimate based on a sampling frequency of 0.1 m). Time consumption was recorded for both methods (TM—cycle time of manual measurement, TS1R1—cycle time of digital measurement for Subject 1 during replication 1, TS1R2—cycle time of digital measurement for Subject 1 during replication 2, TS2R1—cycle time of digital measurement for Subject 2 during replication 1, TS2R2—cycle time of digital measurement for Subject 2 during replication 2).

Overall, six main workflows were implemented. The first workflow (REF—Figure 2) involved manually measuring the logs to obtain the large-end diameter (Dl), small-end diameter (Ds), diameter at the middle (Dm), and log length (L). This workflow was conducted once per log, starting from the same end of the log. The collected data was recorded on paper sheets and then manually transferred to a Microsoft Excel worksheet.

Digital measurement workflows were implemented as illustrated in Figure 2. For each subject and replication, the TS app was used to detect the diameter at the first end of the log. The subject then moved along the log while scanning and concluded the task by detecting the diameter at the second end. In all digital measurement workflows, movement along the log was performed at a constant pace, although there were variations in movement speed among the subjects. After each measurement, data were processed and saved locally, then transferred to a personal computer via AirDrop at the end of the field test. For each subject and replication, two datasets were created and saved as Microsoft Excel files, corresponding to estimates based on the algorithm’s sampling density at 0.5 m (VAPP1) and 0.1 m (VAPP2), respectively. This resulted in eight datasets named based on sampling density (VAPP), subject (S), and replication (R), as shown in Figure 2. Although the digital volume estimates were collected with a precision of more than ten digits, for consistency, these estimates were included in the database rounded to five digits.

Time consumption for manual (TM) and digital (TS1R1, TS1R2, TS2R1, TS2R2) log measurements was assessed using the snap-back chronometry method [24]. The chronometer was set to zero before measuring each log. For manual measurements, the team verbally communicated their intention to start, at which point the chronometer was activated. This communication always occurred after the team arrived at a given log. Upon completing the measurement of a log, they verbally indicated the end of the process, the chronometer was stopped, and the time was recorded on a paper sheet. A similar procedure was followed to collect time consumption for digital measurements. Each subject communicated verbally their intention to start the measurement process and used the app interface to initiate it, at which point the chronometer was activated. Once a measurement was completed, including data processing and saving on the smartphone, the subject verbally indicated the end of the process, and the chronometer was stopped. All time measurements were recorded to the nearest second by a researcher using the chronometer app on an iPhone 13 Pro Max.

For clarity, this study used the following abbreviations: VAPP1_S1R1—volume estimate using the algorithm sampling density of 0.5 m for data collected by Subject 1 during replication 1, VAPP2_S1R1—volume estimate using the algorithm sampling density of 0.1 m for data collected by Subject 1 during replication 1, VAPP1_S1R2—volume estimate using the algorithm sampling density of 0.5 m for data collected by Subject 1 during replication 2, VAPP2_S1R2—volume estimate using the algorithm sampling density of 0.1 m for data collected by Subject 1 during replication 2, VAPP1_S2R1—volume estimate using the algorithm sampling density of 0.5 m for data collected by Subject 2 during replication 1, VAPP2_S2R1—volume estimate using the algorithm sampling density of 0.1 m for data collected by Subject 2 during replication 1, VAPP1_S2R2—volume estimate using the algorithm sampling density of 0.5 m for data collected by Subject 2 during replication 2, VAPP2_S2R2—volume estimate using the algorithm sampling density of 0.1 m for data collected by Subject 2 during replication 2, C—slope of the regression through the origin, and R^2^—coefficient of determination.

### 2.4. Data Processing

Data processing steps were primarily supported by Microsoft Excel. In the first step, all data were paired according to the log identification number. Volume estimates produced for each subject, replication, and sampling density were manually extracted from the .json files and entered into the database. Among other variables, the final database contained paired data for all variables described in Figure 2. Manually collected biometric data were used to compute the volume of each log based on Huber’s (VH) and Smalian’s (VS) formulae. The time consumption of digital measurements (TS1R1, TS1R2, TS2R1, TS2R2) was averaged for each log, resulting in an estimate (AMT) rounded to the nearest second. Subsequently, all data were prepared for statistical analysis according to the objectives of this study. This step included organizing data into separate sheets to meet the requirements of each statistical workflow, as described in Figures S2–S7 (see Supplementary Materials).

### 2.5. Data Analysis

Data analysis was structured into separate workflows (Figures S2–S7, Supplementary Materials) designed in accordance with the objectives of this study. Descriptive statistics, including those showing the distribution of data (Table S1 and Figure S2, Supplementary Materials), were developed to characterize the REF dataset. All variables were subjected to a normality check using the Shapiro–Wilk test [25]. Subsequently, relevant statistical descriptors such as the minimum, maximum, mean, standard deviation, and median values were estimated.

For the first four objectives of this study, the statistical workflows were similar and included checking the paired data for heteroscedasticity using the Breusch–Pagan and White tests [26,27] (Figures S3–S6, Supplementary Materials), assessing trends in the data using the regression through the origin [28], evaluating agreement using Bland and Altman’s method [29], and characterizing the magnitude of differences using bias (hereafter called Bias [30,31]), mean absolute error (hereafter called MAE [32]), and root mean squared error (hereafter called RMSE [33,34]). The statistical workflow for the fourth objective diverged slightly, as the agreement assessment using Bland and Altman’s method was omitted due to the extensive number of comparisons required and the limited space of this paper. However, trends in data and error metrics were computed and reported for all eight possible comparisons.

For the last objective of this study, a normality check using the Shapiro–Wilk test was first conducted on the compared variables (TM and AMT). Next, the potential effects of log biometrics on time consumption were evaluated using least squares ordinary regression for both variables. The log biometrics considered in this analysis were manually collected. Because the data failed the normality test, the nonparametric Mann–Whitney comparison test [35,36,37] was subsequently implemented to determine whether there were statistically significant differences in time consumption between the data measurement methods. Descriptive statistics for this workflow were reported similarly to those characterizing the log biometrics obtained through manual measurement.

Statistical tests and the development of descriptive statistics were conducted using the Real Statistics add-in for Excel [38]. Trends analyzed included those checked by regression through the origin as well as those utilizing ordinary least squares regression, all performed using the functionalities of Microsoft Excel. The results were primarily presented in tabular form, highlighting the main features and interpretations of the regression equations, and were accompanied by scatterplots displaying the data against the identity (1:1) lines. Where relevant, differences between the datasets were included in the trend scatterplots.

Bland–Altman analysis was performed by integrating the data computing and processing functionalities of Microsoft Excel with its capabilities for developing scatterplots. Error (difference) metrics were estimated using commonly known formulas based on data organized in advance for each statistical workflow. For instance, signed, absolute, and squared differences between each pair of compared variables were computed in advance. For convenience, where relevant, these metrics were included in the Bland–Altman plots and reported alongside the upper and lower limits of agreement based on two standard deviations of the data.

## 3. Results

### 3.1. Log Biometrics Based on Manual Measurement

The dataset obtained through manual measurement consisted of 155 observations (logs) and was characterized by a high diversity of the biometrics, particularly in the estimated volumes. The coefficients of variation computed for the Ds, Dl, and Dm variables exhibited similar values in the range of 27% to 29%. By contrast, log length (L) showed reduced variability, with a coefficient of variation of 5%. The volume estimates had coefficients of variation ranging from 54% to 57%. Table S1 (Supplementary Materials) presents the detailed descriptive statistics of the REF dataset, indicating that the volume estimates based on Huber’s formula ranged from approximately 0.05 to 0.76 m^3^, while those based on Smalian’s formula ranged from about 0.07 to 0.96 m^3^. The mean values of the estimates were identical (0.263 m^3^), while the median values were also similar (0.233 m^3^ for VH and 0.235 m^3^ for VS). All variables failed the normality test, although some were close to a standard normal distribution (Figure S1, Supplementary Materials). The results from the heteroscedasticity tests indicate that the data were heteroscedastic (Table S2, Supplementary Materials). Moreover, the analysis of the trends (Figure S9, Supplementary Materials), along with the Bland–Altman plot and error metrics (Figure S10, Supplementary Materials), indicates a high level of agreement in the data.

### 3.2. Trends, Agreement, and Differences in the Algorithm’s Estimates

Table 1 summarizes the values of the slopes (C) and coefficients of determination (R^2^) derived from the trend models developed using regression through the origin for both the actual values and the differences between the actual values. The results of heteroscedasticity tests indicate that most of the data were heteroscedastic, which suggests the presence of proportional bias (see Table S3, Supplementary Materials). The slopes (C) and coefficients of determination (R^2^) for the actual values demonstrate a high level of agreement, as their values were close to 1 in both cases. In regression through the origin, a slope value close to 1 suggests that the data points closely align with the identity line (see Figures S11–S14, Supplementary Materials), whereas a coefficient of determination equal to 1 indicates that 100% of the variability in the dependent variable can be explained by the variability in the independent variable.

**Figure 3:**
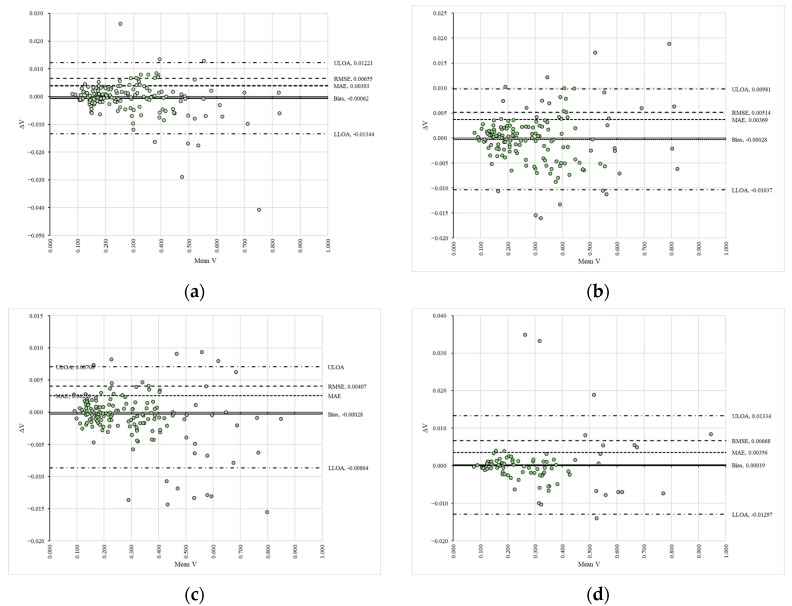
Bland–Altman plots of the compared variables for the algorithm’s estimates: (**a**) comparison between VAPP1_S1R1 and VAPP2_S1R1; (**b**) comparison between VAPP1_S1R2 and VAPP2_S1R2; (**c**) comparison between VAPP1_S2R1 and VAPP2_S2R1; (**d**) comparison between VAPP1_S2R2 and VAPP2_S2R2.

The regression models developed for the differences in the data reveal two key points: the slope was consistently close to zero, indicating a constant trend line, and the coefficients of determination suggested a poor explanation of variability (Table 1). These trends are clearly observable in Figures S11–S14 (Supplementary Materials), which illustrate a general lack of significant differences in the data. Similar findings are presented in Figure 3, where the Bland–Altman plots indicate a high degree of agreement in the estimates, as the data points fall within the agreement limits and show a narrow range of values for these limits.

In these plots (Figure 3), the signed differences (ΔV) between the reference (VAPP1_S1R1—panel (a), VAPP1_S1R2—panel (b), VAPP1_S2R1—panel (c), VAPP1_S2R2—panel (d)) and compared (VAPP2_S1R1—panel (a), VAPP2_S1R2—panel (b), VAPP2_S2R1—panel (c), VAPP2_S2R2—panel (d)) data are plotted against their mean values (Mean V) in a space delimited by the upper (ULOA) and lower (LLOA) limits of agreement, along with figures such as the fixed bias (Bias), mean absolute error (MAE) and root mean squared error (RMSE). Moreover, the error metrics estimated for the compared datasets indicate a systematic bias that remained very low (Bias = −0.00062 to 0.00019), along with low values for mean absolute error (MAE = 0.00258 to 0.00393) and root mean squared error (RMSE = 0.00407 to 0.00668), with the latter suggesting the absence of large-magnitude differences between the datasets.

### 3.3. Trends, Agreement, and Differences in the Replicates

**Figure 4:**
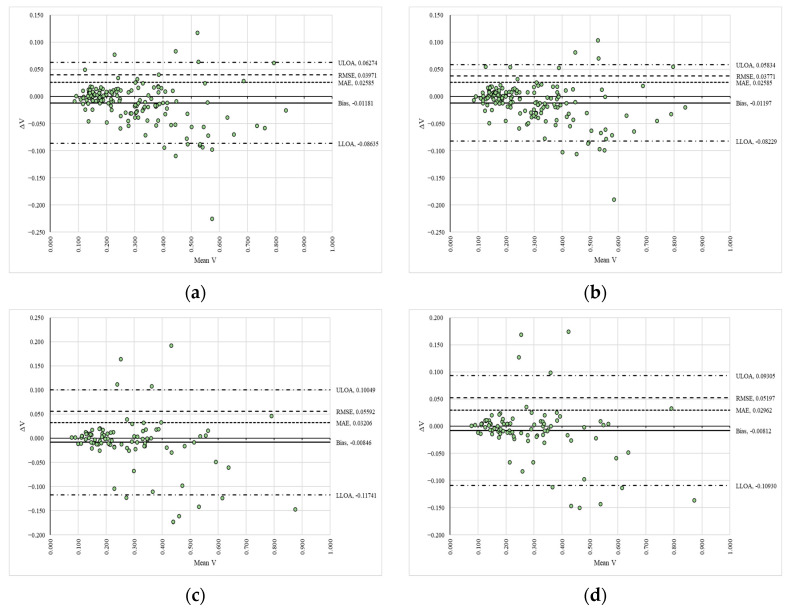
Bland–Altman plots of the compared variables between replicates: (**a**) comparison between VAPP1_S1R1 and VAPP1_S1R2; (**b**) comparison between VAPP2_S1R1 and VAPP2_S1R2; (**c**) comparison between VAPP1_S2R1 and VAPP1_S2R2; (**d**) comparison between VAPP2_S2R1 and VAPP2_S2R2.

The results on the trends, agreement, and differences in the replicates are summarized similarly to those reported in Section 3.2. Table 2 presents the values of the slopes (C) and coefficients of determination (R^2^), while Figure 4 displays the Bland–Altman plots along with the error metrics. Panels (a) to (d) show the signed differences (ΔV) between the reference (VAPP1_S1R1—panel (a), VAPP2_S1R1—panel (b), VAPP1_S2R1—panel (c), VAPP2_S2R1—panel (d)) and compared (VAPP1_S1R2—panel (a), VAPP2_S1R2—panel (b), VAPP1_S2R2—panel (c), VAPP2_S2R2—panel (d)) data plotted against their mean values (Mean V) in a space delimited by the upper (ULOA) and lower (LLOA) limits of agreement, along with figures such as the fixed bias (Bias), mean absolute error (MAE), and root mean squared error (RMSE).

Table S4 (Supplementary Materials) provides the results of the heteroscedasticity tests, and Figures S15–S18 (Supplementary Materials) illustrate the trends in the data. The slopes of the models (C) and coefficients of determination (R^2^) for the actual values indicate good agreement, as their values were close to 1 in both cases. However, the differences from the unit values were greater, due to higher dispersion in the data (see Figures S15–S18, Supplementary Materials), which is consistent with the results from the Bland–Altman plots, particularly concerning the error metrics, which indicate a higher systematic bias (Bias = −0.00759 to 0.00222), mean absolute error (MAE), and root mean squared error (RMSE).

Contrary to the data reported in Section 3.2, the heteroscedasticity tests indicated the presence of homoscedasticity for the pairs VAPP1_S2R1—VAPP1_S2R2 and VAPP2_S2R1—VAPP2_S2R2 (Table S4, Supplementary Materials), indicating no proportional bias between the measurements. However, it is important to note that these results were based on a smaller sample size consisting of only 89 observations.

### 3.4. Trends, Agreement, and Differences Between the Raters

**Figure 5:**
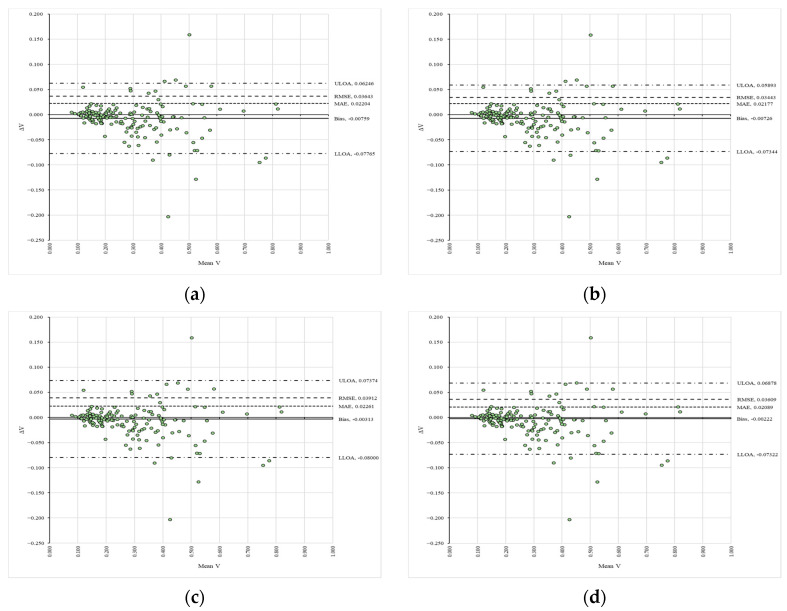
Bland–Altman plots of the compared variables between the raters: (**a**) comparison between VAPP1_S1R1 and VAPP1_S1R2; (**b**) comparison between VAPP2_S1R1 and VAPP2_S1R2; (**c**) comparison between VAPP1_S2R1 and VAPP1_S2R2; (**d**) comparison between VAPP2_S2R1 and VAPP2_S2R2.

Regarding inter-rater consistency, Table 3 summarizes the values of the slopes (C) and coefficients of determination (R^2^), while Figure 5 presents the Bland–Altman plots alongside the error metrics. Panels (a) to (d) show the signed differences (ΔV) between the reference (VAPP1_S1R1—panel (a), VAPP2_S1R1—panel (b), VAPP1_S2R1—panel (c), VAPP2_S2R1—panel (d)) and compared (VAPP1_S1R2—panel (a), VAPP2_S1R2—panel (b), VAPP1_S2R2—panel (c), VAPP2_S2R2—panel (d)) data plotted against their mean values (Mean V) in a space delimited by the upper (ULOA) and lower (LLOA) limits of agreement, along with figures such as the fixed bias (Bias), mean absolute error (MAE), and root mean squared error (RMSE). The supplementary data found in Table S5 (Supplementary Materials) detail the results of the heteroscedasticity tests, and Figures S19–S22 (Supplementary Materials) illustrate the trends in the data. The slopes and coefficients of determination showed the highest deviations from unity for the datasets analyzed for inter-rater consistency, as depicted in Table 3. All data considered in this workflow were found to be heteroscedastic (Table S5, Supplementary Materials). The systematic bias generally exhibited values indicating greater differences (Figure 5), and the mean absolute error (MAE) was slightly higher compared to intra-rater consistency. Furthermore, the root mean squared error (RMSE) suggested the presence of larger-magnitude differences, particularly for the last two compared datasets (Figure 5).

### 3.5. Agreement Between Digital and Manual Data

When comparing the digital estimates to the manually based reference data, the developed models exhibited varying slopes and coefficients of determination, as presented in Table 4. There was similarity in the slopes (C) for the same subject and replication across different sampling densities, and the values for fixed bias (Bias), mean absolute error (MAE), and root mean squared error (RMSE) demonstrated similar behavior (Table 5). Additionally, the scatterplot of the compared datasets is shown in Figure S23 (Supplementary Materials).

### 3.6. Time Efficiency

The time-consumption data did not meet the normality assumption. Therefore, the median value was selected to characterize the central tendency, and the Mann–Whitney nonparametric test was employed for data comparison. The main descriptive statistics for the time-consumption data are presented in Table 6, while the trends in time-consumption variation are illustrated in Figure S24 (Supplementary Materials). Based on the median value, the time consumption for manual measurements was higher by 7 s per log. Additionally, the coefficient of variation (Table 6) indicated greater dispersion in the manual time-consumption dataset compared to the digital measurements. This finding is further supported by the distributions shown in Figure S24, which consistently indicate greater stability in the digital measurement data.

The results from the Mann–Whitney test indicated highly significant statistical differences (*p* = 0.00000, α = 0.05) between the analyzed variables, which were also supported by a very high effect size (r = 82%). According to the descriptive statistics presented in Table 6, the productivity gain when using digital measurements is estimated to be about 38% compared to what can be achieved with manual methods.

## 4. Discussion

This study evaluated the performance of the Tree Scanner (TS) app, a LiDAR-based smartphone tool, during its prototyping phase for log volume estimation. The findings are promising, indicating the app’s potential as an accurate, reliable, and efficient alternative to traditional manual methods, though several considerations and limitations exist. The TS app demonstrated a high degree of accuracy in estimating log volumes when compared to the manual reference data that was evaluated using Huber’s formula. The strong agreement, evidenced by R^2^ values consistently exceeding 0.98 and a low overall RMSE (below 0.067 m^3^), with minor systematic underestimation (Bias approx. −0.0417 m^3^), positions the TS app favorably against other mobile solutions. For instance, Niță and Borz [15] reported that their tested app, based on RANSAC [23] and Poisson Surface Reconstruction [39] algorithms, exhibited proportional bias, particularly for logs outside a specific size range (0.25 to 0.40 m^3^). The TS app, leveraging direct LiDAR scanning for single logs, did not show such pronounced operational bias across different log sizes within our sample, aligning with the general accuracy expectations for LiDAR-based measurements [40,41]. This direct scanning approach contrasts with methods reliant on the geometric assumptions inherent in many manual formulae [42,43] or extensive post-processing. Previous work using LiDAR-equipped iPhones, such as Borz et al. [14], also found high accuracy (95–99%) for individual log diameter and length measurements, corroborating the base capability of the sensor.

However, this study did not specifically test the LiDAR sensor’s ability to accurately capture very-small-diameter logs or highly complex geometries [44]. Several studies show that LiDAR technology inherently has limitations when it comes to capturing very fine details [13,44,45]. Additionally, the current study used sampling densities of 0.1 m and 0.5 m along the log. While both yielded accurate results, the 0.1 m interval provides more data points for shape reconstruction, which could be beneficial for more irregularly shaped logs. Essentially, measurements were conducted under optimal conditions: on a flat, paved, and clean log yard, with the logs spaced apart. Real-world forest operations present significant challenges [46], including uneven terrain, dense understory, or slash, which can create artifacts in point clouds [47,48], varying weather conditions, and mud or debris on logs [46,48,49]. Therefore, the robust performance observed in the controlled settings in this study needs to be validated in typical forest environments.

Moreover, an important characteristic of any measurement tool is its internal consistency [50,51]. The TS app demonstrated excellent internal consistency, with R^2^ values > 0.99 and RMSE values < 0.00407 m^3^ when comparing volume estimates derived from the 0.1 m and 0.5 m sampling densities. This indicates that the app’s underlying algorithms produce highly similar results regardless of the chosen sampling intensity within the tested range [50,51,52]. While the 0.5 m sampling density might be slightly faster in data acquisition, the 0.1 m density captures more detailed surface information. The choice between these may depend on the trade-off between speed and the need for higher-resolution data, especially for logs with significant taper or irregularities [53]. This consistency is vital, as it suggests that users can expect predictable behavior from the app when adjusting this setting, although further study is needed to determine if one interval consistently yields results closer to ground truth across a wider variety of log forms.

Intra-rater reliability, which measures the consistency of measurements taken by the same user on the same objects at different times [54], was also high for the TS app (R^2^ > 0.99 between replications by the same subject). This high consistency is a significant advantage over manual measurements, where slight variations in caliper placement or reading the tape measure can introduce variability between repeated measurements by the same individual [41,44,55], especially if the exact point of diameter sampling is not precisely replicated [44,53,56]. The app’s structured scanning process, capturing data at pre-defined intervals along the log (0.1 m or 0.5 m), likely contributes to this stability by standardizing the data capture methodology between replications for the same user. This suggests that a trained user can repeatedly achieve similar results, enhancing the credibility of the data collected. Forkuo and Borz [57] also reported good reliability for LiDAR-based log measurements, underscoring the potential of this technology.

Similarly, inter-rater reliability, which measures the consistency of results obtained by different users measuring the same objects [58,59], yielded strong agreement, with R^2^ values between 0.9773 and 0.9898. While slightly lower than the intra-rater reliability, which is common, these values indicate that the TS app largely mitigates the influence of individual operator technique. In manual measurements, differences in how operators handle tools, interpret measurement points, and round values can lead to greater discrepancies [41,44]. The app’s guided workflow and automated data capture appear to reduce such operator-specific variability. The study by Forkuo and Borz [57] similarly found that user experience could influence reliability but that LiDAR apps generally offer good inter-rater consistency. The TS app’s performance suggests it can be reliably used by different trained operators with minimal variation in outcomes attributable to personal technique during the scanning process itself [60,61].

In addition, the TS app showed a substantial improvement in time efficiency, with digital measurements requiring a median of 21 s per log compared to 29 s for manual measurements. This represents an estimated productivity gain of approximately 38%. Such efficiency gains are critical in large-scale forest operations, where thousands of logs may need to be measured [62,63]. The Mann–Whitney U test confirmed a highly significant difference in time consumption (*p* < 0.0001), supporting this observation, which is particularly relevant given the non-normal distribution of the time data.

Furthermore, the coefficient of variation for time consumption was considerably lower for digital measurements (10.22%) compared to manual measurements (25.19%). This indicates a more stable and predictable workflow when using the TS app. Traditional manual methods often require a team of at least two, sometimes three, individuals (e.g., for positioning, measuring, and recording) [14,41,44], whereas the TS app can be operated by a single person. When comparing to other technologies, Borz and Proto [64] reported that measuring the diameter and length of single logs took 1.5 min with a different smartphone app and 19 s with a mobile laser scanner, though the latter is a more specialized and costly device. The TS app’s performance for full volume estimation appears competitive, particularly considering that it is a smartphone-based solution. It is important to note that this study did not find evidence that the increased speed of digital measurement compromised its accuracy under the tested conditions.

Several strengths of the Tree Scanner (TS) app have emerged from the evaluation conducted during the prototyping phase. One of the most notable strengths is its accuracy and reliability, which demonstrated a high level of agreement with traditional manual methods. Additionally, the app exhibited strong intra- and inter-rater reliability, reinforcing its dependability in various measurement scenarios. Another significant advantage of the TS app is its time efficiency. The app resulted in a substantial reduction in the measurement time required per log, as well as lower variability in operational time. This improvement indicates that the app can streamline the log measurement process, providing faster results without sacrificing quality. The design of the TS app also prioritizes ease of use and single-operator functionality. It is created for straightforward operation by one person, thereby reducing the labor requirements compared to some manual crews. This feature enhances the app’s practicality and accessibility for users in the field. Furthermore, the TS app delivers direct on-device volume estimation. Users receive instant volume data while in the field, unlike with other apps that only capture raw data for later post-processing. This functionality allows for immediate decision-making based on accurate measurements. In terms of data management, the app offers standardized data capture. The use of LiDAR scanning combined with algorithmic processing minimizes the subjectivity and human error often associated with manual measurements [64], leading to more consistent results. Moreover, the use of this smartphone-based solution for individual log measurements is promising for practical use due to its fast measurement process and lower acquisition costs [65] compared to professional LiDAR scanners. Finally, the app shows significant potential for integration with other technologies [16,66]. It includes functionalities such as Bluetooth connectivity for devices like the HITMAN acoustic tool and RFID readers. It also supports remote server services and species identification features, although these functionalities were not tested in this study. Such capabilities are essential for modern wood traceability systems, as highlighted in previous studies [18,19].

Despite these promising results, this study has limitations because it used a LiDAR-based scanning method [14,15,44], and as a prototype, the app has areas that require further development. One limitation is that this study was conducted in a controlled environment, specifically, in a log yard under optimal conditions. The performance of the app in more challenging forest environments, which are characterized by uneven terrain, poor lighting, occlusions, debris on logs, and adverse weather conditions, has yet to be determined, particularly in terms of accuracy. This represents a critical next step for future research. While the principles of ensuring minimal background noise and a clear line of sight during scanning [45,47,65] were upheld in this study, they may not apply under forest conditions. Another limitation is the limited log variability in the sample used for this study. Although the sample included a range of log sizes, it was restricted to coniferous species from a single location. To fully assess the app’s effectiveness, future testing should encompass diverse species, including hardwoods, and logs with more pronounced irregularities such as sweep, crook, and butt flare. Additionally, future studies should clarify its performance in more challenging environments, for example, when obtaining individual volume measurements among densely packed logs [14,15]. Moreover, there are several untested functionalities within the app that warrant evaluation. Key features designed for enhanced traceability and data management, such as Bluetooth device integration, automatic species identification, and remote server synchronization, were not assessed in this study. Understanding their practical utility and performance is essential for the app’s overall effectiveness. User acceptance and training also pose limitations. Measurements were performed by researchers who were already familiar with the app. Further investigation is needed to assess user acceptance, ease of learning, and the performance of forestry professionals with varying levels of digital literacy [67]. Lastly, while the app tested sampling densities of 0.1 m and 0.5 m, there could be a need to explore a broader range of densities to find an optimal balance between accuracy, processing time, and data storage for various log types.

To build on these findings, future research should prioritize field testing under realistic operational conditions. Expanding the range of tree species and log characteristics, evaluating all integrated functionalities, and conducting user acceptance studies will be essential for the continued development of the TS app.

## 5. Conclusions

This study aimed to evaluate the performance of the prototype Tree Scanner (TS) app, a LiDAR-equipped smartphone application, for direct log volume estimation. The results demonstrate that the TS app is a promising digital measurement tool, exhibiting strong agreement with conventional manual methods while offering a significant improvement in time efficiency. The app maintained consistent accuracy and high reliability across two different sampling densities (0.1 m and 0.5 m) and between different users. The TS app represents a notable advancement by providing instant volume estimates directly on a mobile device, streamlining field data collection. Its ease of use by a single operator and its design integrating features for Bluetooth device connectivity, remote server access, and potential for species identification position it as a valuable component for enhancing forest operations and integrating into comprehensive wood traceability systems. However, as a prototype evaluated under controlled conditions, further research is essential. Future work must focus on assessing the TS app’s performance and robustness in diverse and challenging real-world forest environments and across a wider variety of tree species and log irregularities. Additionally, the practical utility of its extended functionalities and user acceptance by the broader forestry workforce, particularly those with limited experience with digital tools, needs thorough investigation. Addressing these aspects will be essential for realizing the full potential of this app-based smartphone technology in modern forestry.

## Figures and Tables

**Table 1:** Summary statistics of trends in data between the algorithm’s estimates.

Dependent Variable	Independent Variable	Number of Observations	Parameters of the Model for Actual Values	Parameters of the Model for Differences
VAPP2_S1R1	VAPP1_S1R1	155	C = 1.0041 R^2^ = 0.9996	C = −0.0041 R^2^ = 0.0430
VAPP2_S1R2	VAPP1_S1R2	155	C = 1.0006 R^2^ = 0.9998	C = −0.0006 R^2^ = 0.0014
VAPP2_S2R1	VAPP1_S2R1	155	C = 1.0035 R^2^ = 0.9999	C = −0.0035 R^2^ = 0.0844
VAPP2_S2R2	VAPP1_S2R2	89	C = 0.9994 R^2^ = 0.9996	C = −0.0006 R^2^ = 0.0009

**Table 2:** Summary statistics of trends in data between the replicates.

Dependent Variable	Independent Variable	Number of Observations	Parameters of the Model for Actual Values	Parameters of the Model for Differences
VAPP1_S1R2	VAPP1_S1R1	155	C = 1.0231 R^2^ = 0.9888	C = −0.0231 R^2^ = 0.0431
VAPP2_S1R2	VAPP2_S1R1	155	C = 1.0199 R^2^ = 0.9900	C = −0.0199 R^2^ = 0.0364
VAPP1_S2R2	VAPP1_S2R1	89	C = 1.0117 R^2^ = 0.9867	C = −0.0017 R^2^ = 0.0098
VAPP2_S2R2	VAPP2_S2R1	89	C = 1.0082 R^2^ = 0.9886	C = −0.0082 R^2^ = 0.0057

**Table 3:** Summary statistics of trends in data between raters.

Dependent Variable	Independent Variable	Number of Observations	Parameters of the Model for Actual Values	Parameters of the Model for Differences
VAPP1_S2R1	VAPP1_S1R1	155	C = 1.0432 R^2^ = 0.9884	C = −0.0432 R^2^ = 0.1270
VAPP2_S2R1	VAPP2_S1R1	155	C = 1.0431 R^2^ = 0.9898	C = −0.0431 R^2^ = 0.1417
VAPP1_S2R2	VAPP1_S1R2	89	C = 1.0321 R^2^ = 0.9732	C = −0.0458 R^2^ = 0.0737
VAPP2_S2R2	VAPP2_S1R2	89	C = 1.0338 R^2^ = 0.9773	C = −0.0102 R^2^ = 0.0083

**Table 4:** Summary statistics of trends in digital data against the manually based estimates.

Dependent Variable	Independent Variable	Number of Observations	Parameters of the Model for Actual Values
VAPP1_S1R1	VH	155	C = 1.0924 R^2^ = 0.9848
VAPP2_S1R1	VH	155	C = 1.0978 R^2^ = 0.9860
VAPP1_S1R2	VH	155	C = 1.1236 R^2^ = 0.9843
VAPP2_S1R2	VH	155	C = 1.1248 R^2^ = 0.9849
VAPP1_S2R1	VH	155	C = 1.1509 R^2^ = 0.9928
VAPP2_S2R1	VH	155	C = 1.1551 R^2^ = 0.9929
VAPP1_S2R2	VH	89	C = 1.1659 R^2^ = 0.9806
VAPP2_S2R2	VH	89	C = 1.1664 R^2^ = 0.9824

**Table 5:** Error metrics of the digital data when compared with manually based estimates.

Dependent Variable	Independent Variable	Number of Observations	Bias	MAE	RMSE
VAPP1_S1R1	VH	155	−0.0286	0.0353	0.0489
VAPP2_S1R1	VH	155	−0.0292	0.0357	0.0487
VAPP1_S1R2	VH	155	−0.0362	0.0406	0.0559
VAPP2_S1R2	VH	155	−0.0364	0.0405	0.0557
VAPP1_S2R1	VH	155	−0.0404	0.0430	0.0536
VAPP2_S2R1	VH	155	−0.0412	0.0437	0.0546
VAPP1_S2R2	VH	89	−0.0417	0.0495	0.0668
VAPP2_S2R2	VH	89	−0.0416	0.0492	0.0654

**Table 6:** Descriptive statistics of time consumption data.

Variable	Minimum Value	Maximum Value	Mean Value	Median Value	Coefficient of Variation
TM (s)	21	89	31.11	29.00	25.19
ATM (s)	17	29	20.94	21.00	10.22

## Data Availability

The data presented in this study are available from the corresponding author upon reasonable request.

## Supplementary Materials

The following supporting information can be downloaded at: https://www.mdpi.com/article/10.3390/s25185847/s1, Figure S1: QQ-Plots and the relative frequency of biometric data taken by manual measurement. Legend: Panels on the left show the QQ-Plots of the diameter at the small end (Ds, Panel a), diameter at the large end (Dl, Panel c), diameter at the middle (Dm, Panel e), volume estimated by Huber’s formula (VH, Panel g), and volume estimated by Smalian’s formula (VS, Panel i); panels on the right show the relative frequencies in data using histograms for the diameter at the small end (Ds, Panel b), diameter at the large end (Dl, Panel d), diameter at the middle (Dm, Panel f), volume estimated by Huber’s formula (VH, Panel h), and volume estimated by Smalian’s formula (VS, Panel j). Note: Bins’ width and size were computed using the Freedman–Diaconis rule, which is based on interquartile range and the number of observations in the sample; data on log length is not shown in the figure, but it had a bi-modal distribution with modes at about 3.1 and 4.1 m in length; Figure S2: Statistical workflow implemented to characterize the data collected manually. Legend: Ds—diameter at the small end, Dm—diameter at the middle, Dl—diameter at the large end, L—log length, VH—volume estimated by Huber’s formula, VS—volume estimated by Smalian’s formula. Note: All the variables of the REF dataset (Ds, Dm, Dl, and L) as well as those derived (VH, VS), were subjected to a data normality test (Shapiro–Wilk); based on the outcomes of the test, the relevant descriptive statistics were estimated (see also Table S1 and Figure S1); Figure S3: Statistical workflow implemented to characterize the consistency of estimates due to the algorithm’s sampling densities. Legend: VAPP1_S1R1—volume estimate using the algorithm sampling density of 0.5 m for data collected by Subject 1 during replication 1, VAPP2_S1R1—volume estimate using the algorithm sampling density of 0.1 m for data collected by Subject 1 during replication 1, VAPP1_S1R2—volume estimate using the algorithm sampling density of 0.5 m for data collected by Subject 1 during replication 2, VAPP2_S1R2—volume estimate using the algorithm sampling density of 0.1 m for data collected by Subject 1 during replication 2, VAPP1_S2R1—volume estimate using the algorithm sampling density of 0.5 m for data collected by Subject 2 during replication 1, VAPP2_S2R1—volume estimate using the algorithm sampling density of 0.1 m for data collected by Subject 2 during replication 1, VAPP1_S2R2—volume estimate using the algorithm sampling density of 0.5 m for data collected by Subject 2 during replication 2, VAPP2_S2R2—volume estimate using the algorithm sampling density of 0.1 m for data collected by Subject 2 during replication 2. Note: All the pairs of variables were subjected to heteroscedasticity tests (Breusch–Pagan, White). Conventionally, the data from the sampling density of 0.5 m was taken as a reference to check the agreement of estimates by the Blant–Altman method, as well as to compute the relevant difference metrics (Bias, MAE, RMSE). Regression through the origin (RTO) was used to see the trends in data, where the independent variables were those based on a sampling density of 0.5 m. Note that the subject and replication were kept the same in all comparisons; Figure S4: Statistical workflow implemented to characterize the consistency of estimates when considering different replications of the same subject. Legend: VAPP1_S1R1—volume estimate using the algorithm sampling density of 0.5 m for data collected by Subject 1 during replication 1, VAPP2_S1R1—volume estimate using the algorithm sampling density of 0.1 m for data collected by Subject 1 during replication 1, VAPP1_S1R2—volume estimate using the algorithm sampling density of 0.5 m for data collected by Subject 1 during replication 2, VAPP2_S1R2—volume estimate using the algorithm sampling density of 0.1 m for data collected by Subject 1 during replication 2, VAPP1_S2R1—volume estimate using the algorithm sampling density of 0.5 m for data collected by Subject 2 during replication 1, VAPP2_S2R1—volume estimate using the algorithm sampling density of 0.1 m for data collected by Subject 2 during replication 1, VAPP1_S2R2—volume estimate using the algorithm sampling density of 0.5 m for data collected by Subject 2 during replication 2, VAPP2_S2R2—volume estimate using the algorithm sampling density of 0.1 m for data collected by Subject 2 during replication 2. Note: All the pairs of variables were subjected to heteroscedasticity tests (Breusch–Pagan, White). Conventionally, the data from the sampling density of 0.5 m was taken as a reference to check the agreement in estimates by the Blant–Altman method, as well as to compute the relevant difference metrics (Bias, MAE, RMSE). Regression through the origin (RTO) was used to see the trends in data, where the independent variables were those based on a sampling density of 0.5 m. Note that the sampling density and the subject were kept the same in all comparisons; Figure S5: Statistical workflow implemented to characterize the inter-rater consistency of estimates. Legend: VAPP1_S1R1—volume estimate using the algorithm sampling density of 0.5 m for data collected by Subject 1 during replication 1, VAPP2_S1R1—volume estimate using the algorithm sampling density of 0.1 m for data collected by Subject 1 during replication 1, VAPP1_S1R2—volume estimate using the algorithm sampling density of 0.5 m for data collected by Subject 1 during replication 2, VAPP2_S1R2—volume estimate using the algorithm sampling density of 0.1 m for data collected by Subject 1 during replication 2, VAPP1_S2R1—volume estimate using the algorithm sampling density of 0.5 m for data collected by Subject 2 during replication 1, VAPP2_S2R1—volume estimate using the algorithm sampling density of 0.1 m for data collected by Subject 2 during replication 1, VAPP1_S2R2—volume estimate using the algorithm sampling density of 0.5 m for data collected by Subject 2 during replication 2, VAPP2_S2R2—volume estimate using the algorithm sampling density of 0.1 m for data collected by Subject 2 during replication 2. Note: All the pairs of variables were subjected to heteroscedasticity tests (Breusch–Pagan, White). Conventionally, the data from the sampling density of 0.5 m was taken as a reference to check the agreement in estimates by the Blant–Altman method, as well as to compute the relevant difference metrics (Bias, MAE, RMSE). Regression through the origin (RTO) was used to see the trends in data, where the independent variables were those based on a sampling density of 0.5 m. Note that the sampling density and the replication were kept the same in all comparisons; Figure S6: Statistical workflow implemented to characterize the agreement between digital and manual estimates. Legend: Ds—diameter at the small end, Dm—diameter at the middle, Dl—diameter at the large end, L—log length, VH—volume estimated by Huber’s formula, VS—volume estimated by Smalian’s formula, VAPP1_S1R1—volume estimate using the algorithm sampling density of 0.5 m for data collected by Subject 1 during replication 1, VAPP2_S1R1—volume estimate using the algorithm sampling density of 0.1 m for data collected by Subject 1 during replication 1, VAPP1_S1R2—volume estimate using the algorithm sampling density of 0.5 m for data collected by Subject 1 during replication 2, VAPP2_S1R2—volume estimate using the algorithm sampling density of 0.1 m for data collected by Subject 1 during replication 2, VAPP1_S2R1—volume estimate using the algorithm sampling density of 0.5 m for data collected by Subject 2 during replication 1, VAPP2_S2R1—volume estimate using the algorithm sampling density of 0.1 m for data collected by Subject 2 during replication 1, VAPP1_S2R2—volume estimate using the algorithm sampling density of 0.5 m for data collected by Subject 2 during replication 2, VAPP2_S2R2—volume estimate using the algorithm sampling density of 0.1 m for data collected by Subject 2 during replication 2. Note: All the pairs of variables were subjected to heteroscedasticity tests (Breusch–Pagan, White). Conventionally, VH was taken as the independent variable in the regression through the origin (RTO), which was used to see the trends in data and as a reference to estimate the differences in the Bias, MAE, and RMSE metrics. Note that all the possible comparisons were taken into account; Figure S7: Statistical workflow implemented to compare the methods’ time efficiency. Legend: TM—cycle time of manual measurement, TS1R1—cycle time of digital measurement taken by Subject 1 during replication 1, TS1R2—cycle time of digital measurement taken by Subject 1 during replication 2, TS2R1—cycle time of digital measurement taken by Subject 2 during replication 1, TS2R2—cycle time of digital measurement taken by Subject 2 during replication 2, AMT—average digital measurement cycle time. Note: AMT was computed as the mean value rounded to the nearest second of TS1R1, TS1R2, TS2R1, and TS2R2. All the variables were subjected to data normality tests (Shapiro–Wilk) to decide what statistics could be used to characterize the central tendency as well as to select the right type of statistical comparison test; Figure S8: Frequency of differences between VH and VS plotted against a normal density curve; Figure S9: Trend of VS (green dots) and of the differences between the estimates (ΔV, brown dots) as a function of VH, plotted against the identity (1:1) line shown as the black dashed line. Legend: VH—volume estimated by Huber’s formula, VS—volume estimated by Smalian’s formula, ΔV—signed difference between VH and VS. Note: The sample contained 155 logs; Figure S10: Bland–Altman plot showing the agreement of estimates between VH and VS. Legend: ΔV—signed differences between volume estimates, Mean V—average value of volume estimates, ULOA—upper limit of agreement, RMSE—root mean squared error, MAE—mean absolute error, Bias—bias, LLOA—lower limit of agreement. Note: There was good agreement when considering two standard deviations to compute the limits of agreement; the Bias was close to zero, meaning that there was low systematic bias. However, the mean absolute error was close to 0.02, and some observations large in magnitude led to an RMSE value of 0.03; Figure S11: Scatterplot of VAPP1_S1R1 against VAPP2_S1R1. Legend: Green dots stand for the paired values of VAPP1_S1R1 and VAPP2_S1R1 and are plotted against the identity (1:1) line showed as black dashed line; brown dots stand for the signed difference between VAPP1_S1R1 and VAPP2_S1R1. Note: The sample contained 155 observations; Figure S12: Scatterplot of VAPP1_S1R2 against VAPP2_S1R2. Legend: Green dots stand for the paired values of VAPP1_S1R2 and VAPP2_S1R2 and are plotted against the identity (1:1) line showed as black dashed line; brown dots stand for the signed difference between VAPP1_S1R2 and VAPP2_S1R2. Note: The sample contained 155 observations; Figure S13: Scatterplot of VAPP1_S2R1 against VAPP2_S2R1. Legend: Green dots stand for the paired values of VAPP1_S2R1 and VAPP2_S2R1 and are plotted against the identity (1:1) line showed as black dashed line; brown dots stand for the signed differences between VAPP1_S2R1 and VAPP2_S2R1. Note: The sample contained 155 observations; Figure S14: Scatterplot of VAPP1_S2R2 against VAPP2_S2R2. Legend: Green dots stand for the paired values of VAPP1_S2R2 and VAPP2_S2R2 and are plotted against the identity (1:1) line showed as black dashed line; brown dots stand for the signed differences between VAPP1_S2R2 and VAPP2_S2R2. Note: The sample contained 86 observations; Figure S15: Scatterplot of VAPP1_S1R1 against VAPP1_S1R2. Legend: Green dots stand for the paired values of VAPP1_S1R1 and VAPP1_S1R2 and are plotted against the identity (1:1) line showed as black dashed line; brown dots stand for the signed difference between VAPP1_S1R1 and VAPP1_S1R2. Note: The sample contained 155 observations; Figure S16: Scatterplot of VAPP2_S1R1 against VAPP2_S1R2. Legend: Green dots stand for the paired values of VAPP2_S1R1 and VAPP2_S1R2 and are plotted against the identity (1:1) line showed as black dashed line; brown dots stand for the signed difference between VAPP2_S1R1 and VAPP2_S1R2. Note: The sample contained 155 observations; Figure S17: Scatterplot of VAPP1_S2R1 against VAPP1_S2R2. Legend: Green dots stand for the paired values of VAPP1_S2R1 and VAPP1_S2R2 and are plotted against the identity (1:1) line showed as black dashed line; brown dots stand for the signed difference between VAPP1_S2R1 and VAPP1_S2R2. Note: The sample contained 89 observations; Figure S18: Scatterplot of VAPP2_S2R1 against VAPP2_S2R2. Legend: Green dots stand for the paired values of VAPP2_S2R1 and VAPP2_S2R2 and are plotted against the identity (1:1) line showed as black dashed line; brown dots stand for the signed difference between VAPP2_S2R1 and VAPP2_S2R2. Note: The sample contained 89 observations; Figure S19: Scatterplot of VAPP1_S1R1 against VAPP1_S2R1. Legend: Green dots stand for the paired values of VAPP1_S1R1 and VAPP1_S2R1 and are plotted against the identity (1:1) line showed as black dashed line; brown dots stand for the signed difference between VAPP1_S1R1 and VAPP1_S2R1. Note: The sample contained 155 observations; Figure S20: Scatterplot of VAPP2_S1R1 against VAPP2_S2R1. Legend: Green dots stand for the paired values of VAPP2_S1R1 and VAPP2_S2R1 and are plotted against the identity (1:1) line showed as black dashed line; brown dots stand for the signed difference between VAPP2_S1R1 and VAPP2_S2R1. Note: The sample contained 155 observations; Figure S21: Scatterplot of VAPP1_S1R2 against VAPP1_S2R2. Legend: Green dots stand for the paired values of VAPP1_S1R2 and VAPP1_S2R2 and are plotted against the identity (1:1) line showed as black dashed line; brown dots stand for the signed difference between VAPP1_S1R2 and VAPP1_S2R2. Note: The sample contained 89 observations; Figure S22: Scatterplot of VAPP2_S1R2 against VAPP2_S2R2. Legend: Green dots stand for the paired values of VAPP2_S1R2 and VAPP2_S2R2 and are plotted against the identity (1:1) line showed as black dashed line; brown dots stand for the signed difference between VAPP2_S1R2 and VAPP2_S2R2. Note: The sample contained 89 observations; Figure S23: Scatterplot of VAPP variables against VH. Note: Shapes of various colors stand for the paired values of the eight datasets (see the figure legend) against the VH estimates and are plotted against the identity (1:1) line showed as black dashed line. Note: The sample contained 89 observations when using VAPP1_S2R2 and VAPP2_S3R2 datasets; Figure S24: Dependence of time consumption for manual (TM) and digital (AMT) measurements on variability of log biometrics collected manually. Legend: TM—cycle time for manual measurement, AMT—cycle time for digital measurement, Ds—diameter at the small end, Dl—diameter at the large end, Dm—diameter at the middle, L—log length, VH—volume estimated by Huber’s formula; Table S1: Summary statistics of log biometric data taken by manual measurement; Table S2: Results of heteroscedasticity tests; Table S3: Results of heteroscedasticity tests; Table S4: Results of heteroscedasticity tests; Table S5: Results of heteroscedasticity tests.
