# Supplementary material for: Accuracy of a Novel Smartphone-Based Log Measurement App in the Prototyping Phase
Source: Sensors (Basel). 2025 Sep 19;25(18):5847. doi: 10.3390/s25185847 (PMC12473548; doi:10.3390/s25185847)
Supplement: Supplementary file 1 [file sensors-25-05847-s001.zip › sensors-3841741-supplementary.pdf]

sensors

MDPI

Supplementary Materials

Performance of a Novel Smartphone-Based Log Measurement App in the Prototyping Phase

By Mirella Elias[1], Gabriel Osei Forkuo[1], Gianni Picchi[2], Carla Nati[2], Stelian Alexandru Borz[1,*]
[1] Department of Forest Engineering, Forest Management Planning and Terrestrial Measurements, Faculty of Silviculture and Forest Engineering, Transilvania University of Brasov, Şirul Beethoven 1, 500123, Brasov, Romania, mirella.elias@unitbv.ro (M.E.), gabriel.forkuo@unitbv.ro (G.O.F.), stelian.borz@unitbv.ro (S.A.B.).
[2] Institute of Bioeconomy, National Research Council, Via Madonna del Piano 10, 50019, Sesto Fiorentino, Italy, gianni.picchi@cnr.it (G.P.), carla.nati@cnr.it (C.N.).
*Corresponding author: stelian.borz@unitbv.ro

1. Summary statistics of the log biometric data taken by manual measurement

Table S1. Summary statistics of log biometric data taken by manual measurement.

Parameter (measurement unit)	Minimum value	Maximum value	Mean value	Standard deviation value	Median value
Ds (cm)	12.90	48.10	25.76	±0.61	25.00
Dl (cm)	17.40	61.10	29.63	±0.64	28.80
Dm (cm)	13.10	48.90	27.86	±0.59	27.00
L (m)	3.08	4.31	4.04	±0.01	4.08
VH (m^3)	0.054	0.761	0.263	±0.011	0.233
VS (m^3)	0.073	0.962	0.263	±0.012	0.235

Legend: Ds—diameter at the small end, Dl—diameter at the large end, Dm—diameter at the middle, L—log length, VH—volume estimated by Huber's formula, VS—volume estimated by Smalian's formula. Note: The sample contained 155 logs, and none of the variables passed the normality test.

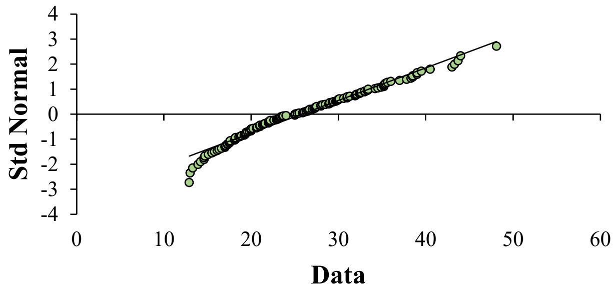
Std Normal
Data
4 3 2 1 0 -1 -2 -3 -4
0 10 20 30 40 50 60

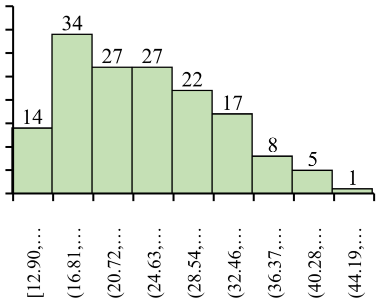
14 34 27 27 22 17 8 5 1
[12.90,… (16.81,… (20.72,… (24.63,… (28.54,… (32.46,… (36.37,… (40.28,… (44.19,…

a

b

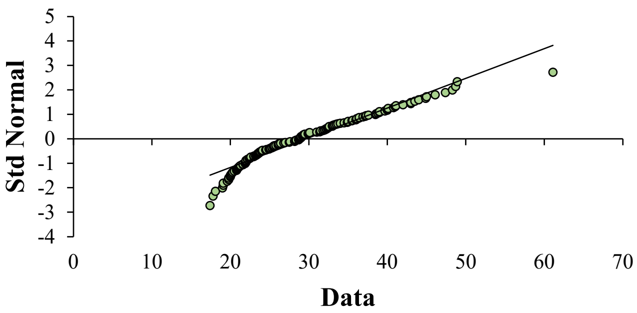
Std Normal
Data
5 4 3 2 1 0 -1 -2 -3 -4
0 10 20 30 40 50 60 70

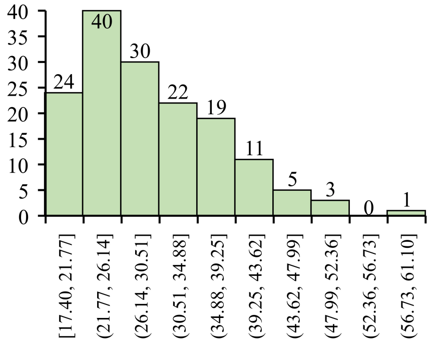
40 35 30 25 20 15 10 5 0
24 40 30 22 19 11 5 3 0 1
[17.40, 21.77] (21.77, 26.14] (26.14, 30.51] (30.51, 34.88] (34.88, 39.25] (39.25, 43.62] (43.62, 47.99] (47.99, 52.36] (52.36, 56.73] (56.73, 61.10]

c

d

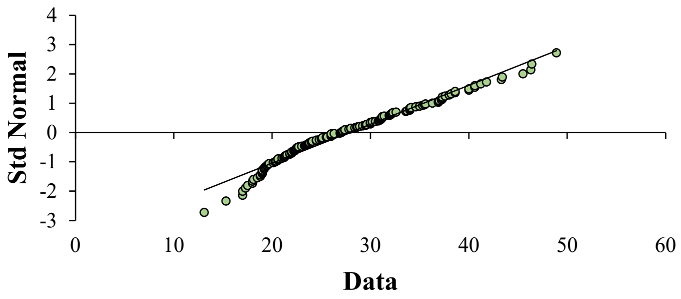
Std Normal
Data
4 3 2 1 0 -1 -2 -3
0 10 20 30 40 50 60

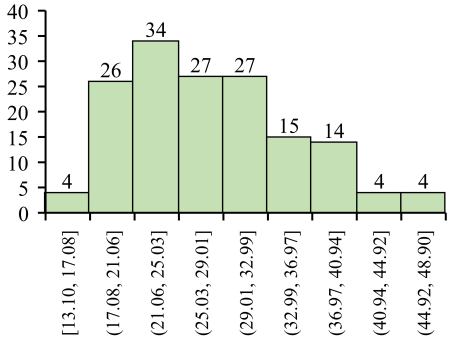
40 35 30 25 20 15 10 5 0
4 26 34 27 27 15 14 4 4
[13.10, 17.08] (17.08, 21.06] (21.06, 25.03] (25.03, 29.01] (29.01, 32.99] (32.99, 36.97] (36.97, 40.94] (40.94, 44.92] (44.92, 48.90]

e

f

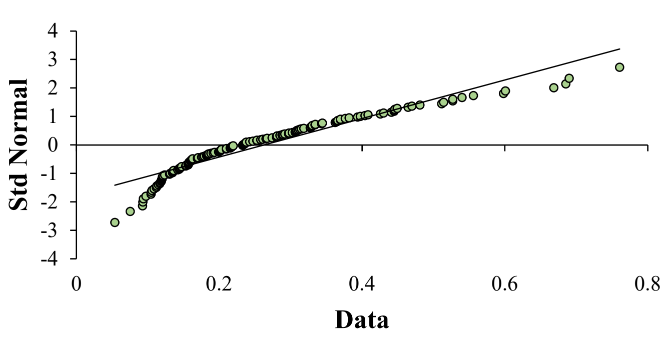
Std Normal
Data
4 3 2 1 0 -1 -2 -3 -4
0 0.2 0.4 0.6 0.8

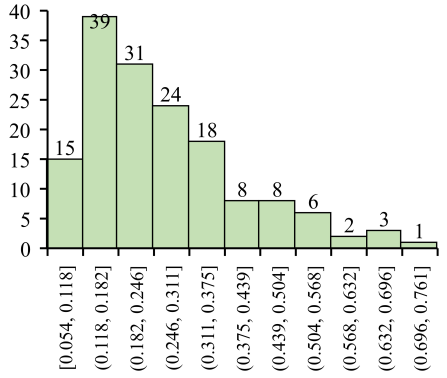
40 35 30 25 20 15 10 5 0
15 39 31 24 18 8 8 6 2 3 1
[0.054, 0.118] (0.118, 0.182] (0.182, 0.246] (0.246, 0.311] (0.311, 0.375] (0.375, 0.439] (0.439, 0.504] (0.504, 0.568] (0.568, 0.632] (0.632, 0.696] (0.696, 0.761]

g

h

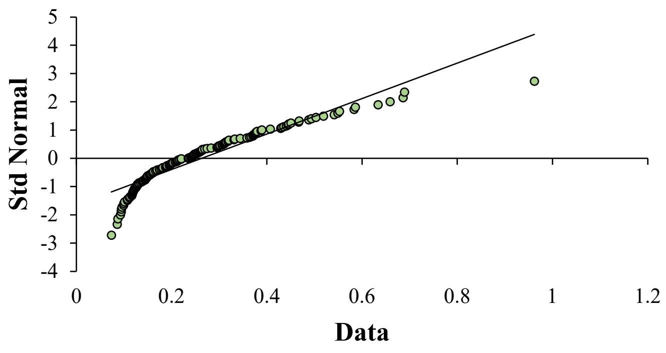

i

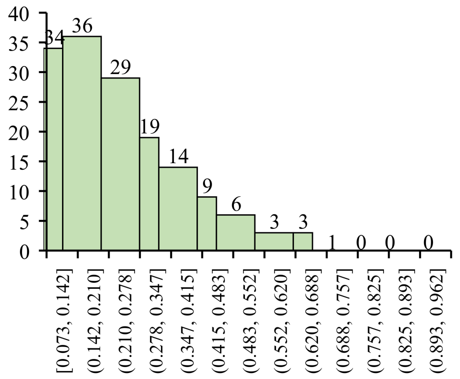

j

Figure S1. QQ-Plots and the relative frequency of biometric data taken by manual measurement. Legend: Panels on the left show the QQ-Plots of the diameter at the small end (Ds, Panel a), diameter at the large end (Dl, Panel c), diameter at the middle (Dm, Panel e), volume estimated by Huber's formula (VH, Panel g), and volume estimated by Smalian's formula (VS, Panel i); panels on the right show the relative frequencies in data using histograms for the diameter at the small end (Ds, Panel b), diameter at the large end (Dl, Panel d), diameter at the middle (Dm, Panel f), volume estimated by Huber's formula (VH, Panel h), and volume estimated by Smalian's formula (VS, Panel j). Note: Bins' width and size were computed using the Freedman–Diaconis rule, which is based on inter-quartile range and the number of observations in the sample; data on log length is not shown in the figure, but it had a bi-modal distribution with modes at about 3.1 and 4.1 meters in length.

2. Statistical workflows implemented to develop the descriptive statistics, check the trends and agreement in data, and characterize the differences in data

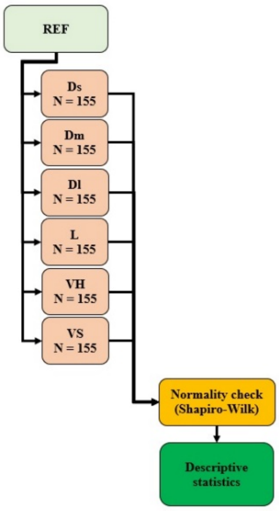

Figure S2. Statistical workflow implemented to characterize the data collected manually. Legend: Ds—diameter at the small end, Dm—diameter at the middle, Dl—diameter at the large end, L—log length, VH—volume estimated by Huber's formula, VS—volume estimated by Smalian's formula. Note: All the variables of the REF dataset (Ds, Dm, Dl, and L) as well as those derived (VH, VS), were subjected to a data normality test (Shapiro–Wilk); based on the outcomes of the test, the relevant descriptive statistics were estimated (see also Table S1 and Figure S1).

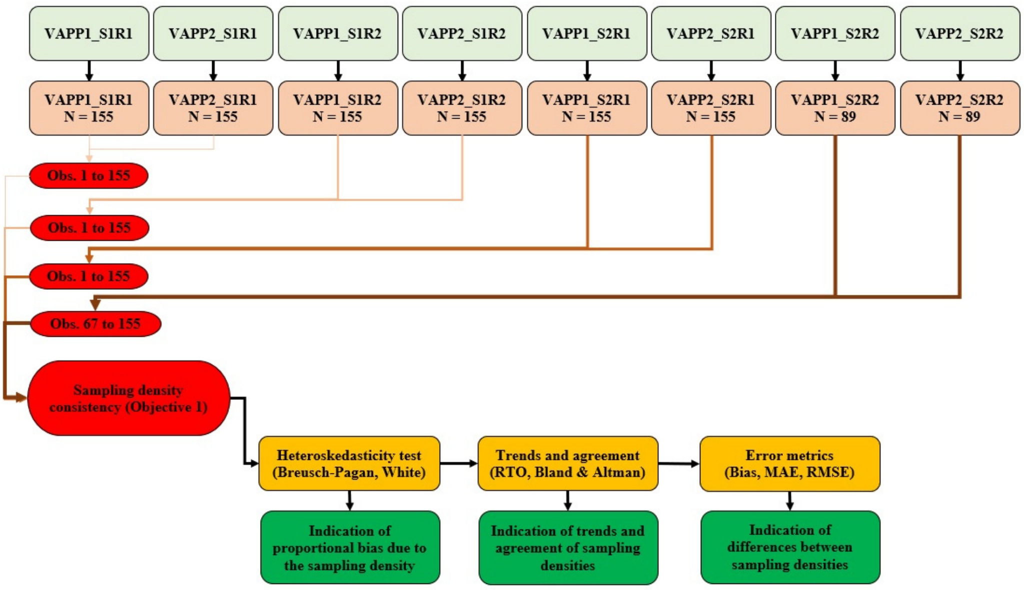

Figure S3. Statistical workflow implemented to characterize the consistency of estimates due to the algorithm's sampling densities. Legend: VAPP1_S1R1—volume estimate using the algorithm sampling density of 0.5 m for data collected by Subject 1 during replication 1, VAPP2_S1R1—volume estimate using the algorithm sampling density of 0.1 m for data collected by Subject 1 during replication 1, VAPP1_S1R2—volume estimate using the algorithm sampling density of 0.5 m for data collected by Subject 1 during replication 2, VAPP2_S1R2—volume estimate using the algorithm sampling density of 0.1 m for data collected by Subject 1 during replication 2, VAPP1_S2R1—volume estimate using the algorithm sampling density of 0.5 m for data collected by Subject 2 during replication 1, VAPP2_S2R1—volume estimate using the algorithm sampling density of 0.1 m for data collected by Subject 2 during replication 1, VAPP1_S2R2—volume estimate using the algorithm sampling density of 0.5 m for data collected by Subject 2 during replication 2, VAPP2_S2R2—volume estimate using the algorithm sampling density of 0.1 m for data collected by Subject 2 during replication 2. Note: All the pairs of variables were subjected to heteroscedasticity tests (Breusch–Pagan, White). Conventionally, the data from the sampling density of 0.5 m was taken as a reference to check the agreement of estimates by the Blant–Altman method, as well as to compute the relevant difference metrics (Bias, MAE, RMSE). Regression through the origin (RTO) was used to see the trends in data, where the independent variables were those based on a sampling density of 0.5 m. Note that the subject and replication were kept the same in all comparisons.

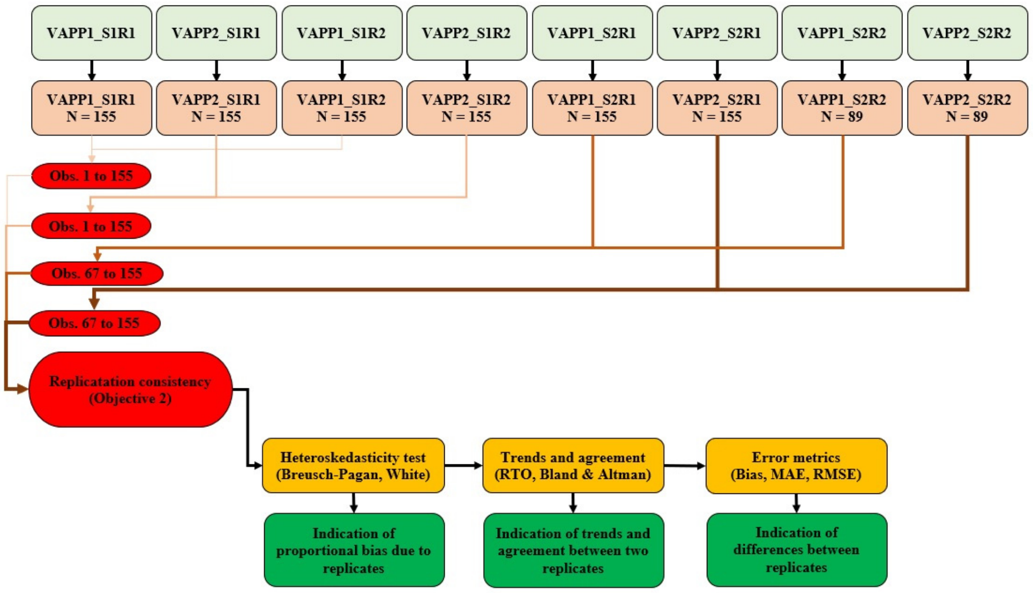

Figure S4. Statistical workflow implemented to characterize the consistency of estimates when considering different replications of the same subject. Legend: VAPP1_S1R1—volume estimate using the algorithm sampling density of 0.5 m for data collected by Subject 1 during replication 1, VAPP2_S1R1—volume estimate using the algorithm sampling density of 0.1 m for data collected by Subject 1 during replication 1, VAPP1_S1R2—volume estimate using the algorithm sampling density of 0.5 m for data collected by Subject 1 during replication 2, VAPP2_S1R2—volume estimate using the algorithm sampling density of 0.1 m for data collected by Subject 1 during replication 2, VAPP1_S2R1—volume estimate using the algorithm sampling density of 0.5 m for data collected by Subject 2 during replication 1, VAPP2_S2R1—volume estimate using the algorithm sampling density of 0.1 m for data collected by Subject 2 during replication 1, VAPP1_S2R2—volume estimate using the algorithm sampling density of 0.5 m for data collected by Subject 2 during replication 2, VAPP2_S2R2—volume estimate using the algorithm sampling density of 0.1 m for data collected by Subject 2 during replication 2. Note: All the pairs of variables were subjected to heteroscedasticity tests (Breusch–Pagan, White). Conventionally, the data from the sampling density of 0.5 m was taken as a reference to check the agreement in estimates by the Blant–Altman method, as well as to compute the relevant difference metrics (Bias, MAE, RMSE). Regression through the origin (RTO) was used to see the trends in data, where the independent variables were those based on a sampling density of 0.5 m. Note that the sampling density and the subject were kept the same in all comparisons.

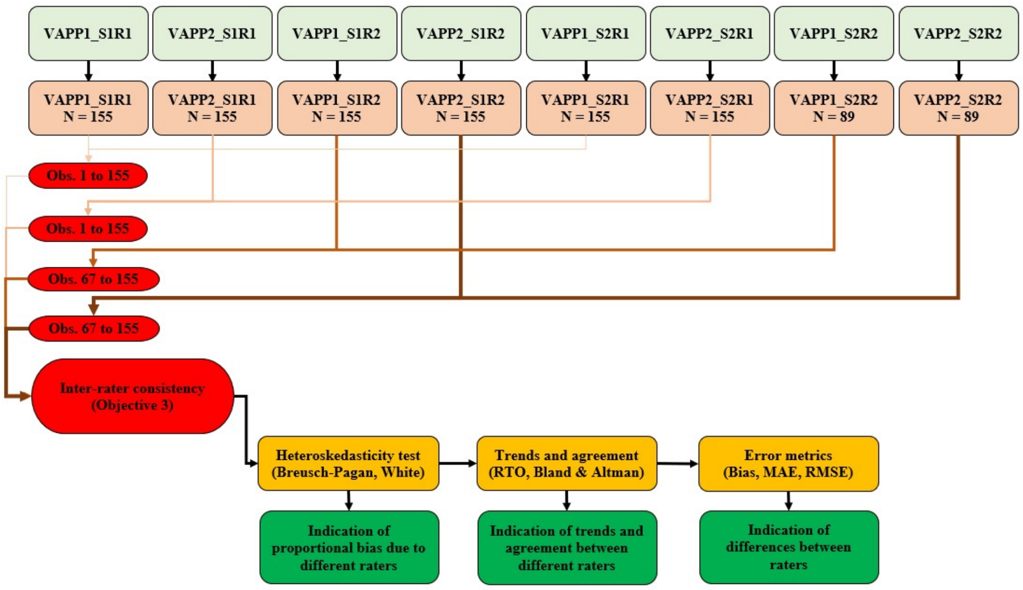

Figure S5. Statistical workflow implemented to characterize the inter-rater consistency of estimates. Legend: VAPP1_S1R1—volume estimate using the algorithm sampling density of 0.5 m for data collected by Subject 1 during replication 1, VAPP2_S1R1—volume estimate using the algorithm sampling density of 0.1 m for data collected by Subject 1 during replication 1, VAPP1_S1R2—volume estimate using the algorithm sampling density of 0.5 m for data collected by Subject 1 during replication 2, VAPP2_S1R2—volume estimate using the algorithm sampling density of 0.1 m for data collected by Subject 1 during replication 2, VAPP1_S2R1—volume estimate using the algorithm sampling density of 0.5 m for data collected by Subject 2 during replication 1, VAPP2_S2R1—volume estimate using the algorithm sampling density of 0.1 m for data collected by Subject 2 during replication 1, VAPP1_S2R2—volume estimate using the algorithm sampling density of 0.5 m for data collected by Subject 2 during replication 2, VAPP2_S2R2—volume estimate using the algorithm sampling density of 0.1 m for data collected by Subject 2 during replication 2. Note: All the pairs of variables were subjected to heteroscedasticity tests (Breusch–Pagan, White). Conventionally, the data from the sampling density of 0.5 m was taken as a reference to check the agreement in estimates by the Blant–Altman method, as well as to compute the relevant difference metrics (Bias, MAE, RMSE). Regression through the origin (RTO) was used to see the trends in data, where the independent variables were those based on a sampling density of 0.5 m. Note that the sampling density and the replication were kept the same in all comparisons.

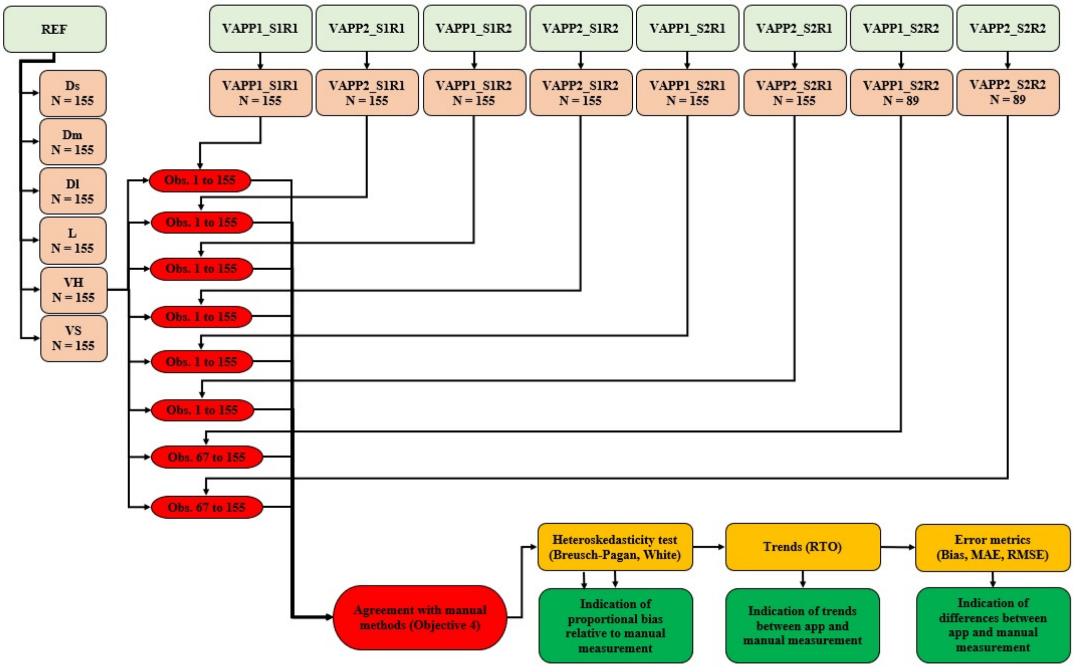

Figure S6. Statistical workflow implemented to characterize the agreement between digital and manual estimates. Legend: Ds—diameter at the small end, Dm—diameter at the middle, Dl—diameter at the large end, L—log length, VH—volume estimated by Huber's formula, VS—volume estimated by Smalian's formula, VAPP1_S1R1—volume estimate using the algorithm sampling density of 0.5 m for data collected by Subject 1 during replication 1, VAPP2_S1R1—volume estimate using the algorithm sampling density of 0.1 m for data collected by Subject 1 during replication 1, VAPP1_S1R2—volume estimate using the algorithm sampling density of 0.5 m for data collected by Subject 1 during replication 2, VAPP2_S1R2—volume estimate using the algorithm sampling density of 0.1 m for data collected by Subject 1 during replication 2, VAPP1_S2R1—volume estimate using the algorithm sampling density of 0.5 m for data collected by Subject 2 during replication 1, VAPP2_S2R1—volume estimate using the algorithm sampling density of 0.1 m for data collected by Subject 2 during replication 1, VAPP1_S2R2—volume estimate using the algorithm sampling density of 0.5 m for data collected by Subject 2 during replication 2, VAPP2_S2R2—volume estimate using the algorithm sampling density of 0.1 m for data collected by Subject 2 during replication 2. Note: All the pairs of variables were subjected to heteroscedasticity tests (Breusch–Pagan, White). Conventionally, VH was taken as the independent variable in the regression through the origin (RTO), which was used to see the trends in data and as a reference to estimate the differences in the Bias, MAE, and RMSE metrics. Note that all the possible comparisons were taken into account.

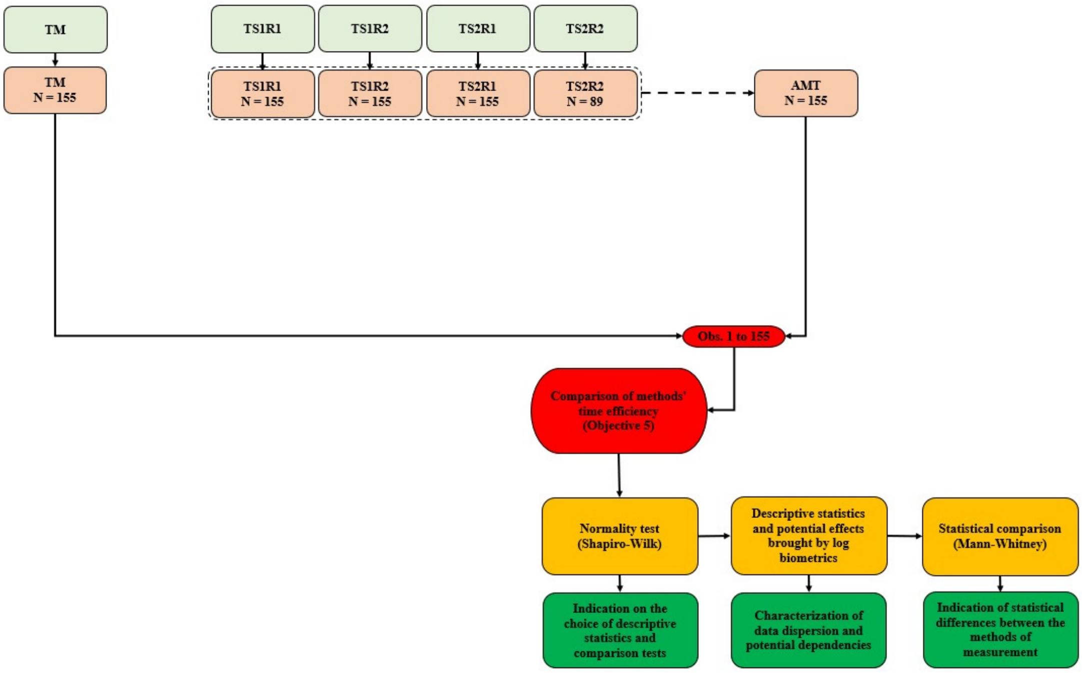

Figure S7. Statistical workflow implemented to compare the methods' time efficiency. Legend: TM—cycle time of manual measurement, TS1R1—cycle time of digital measurement taken by Subject 1 during replication 1, TS1R2—cycle time of digital measurement taken by Subject 1 during replication 2, TS2R1—cycle time of digital measurement taken by Subject 2 during replication 1, TS2R2—cycle time of digital measurement taken by Subject 2 during replication 2, AMT—average digital measurement cycle time. Note: AMT was computed as the mean value rounded to the nearest second of TS1R1, TS1R2, TS2R1, and TS2R2. All the variables were subjected to data normality tests (Shapiro–Wilk) to decide what statistics could be used to characterize the central tendency as well as to select the right type of statistical comparison test.

3. Agreement of volume estimates based on data taken by manual measurement

Table S2. Results of heteroscedasticity tests.

Compared variables	Test	LM Stat	Degrees of freedom	p-value	Outcome
VS, VH	Breusch–Pagan	13.51	1	0.00024	heteroscedastic
VS, VH	White	18.81	2	0.00008	heteroscedastic

Note: The tests run were the Breusch–Pagan and White tests. Breusch–Pagan test is suited for detecting linear heteroscedasticity, whereas White's test can detect other forms of heteroscedasticity.

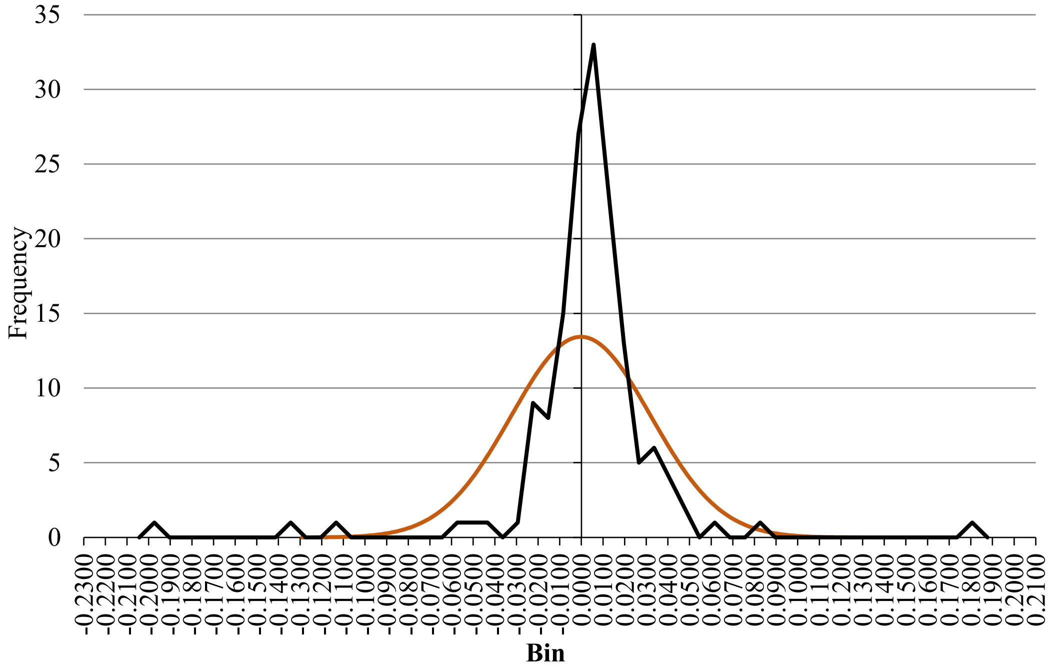

Figure S8. Frequency of differences between VH and VS plotted against a normal density curve.

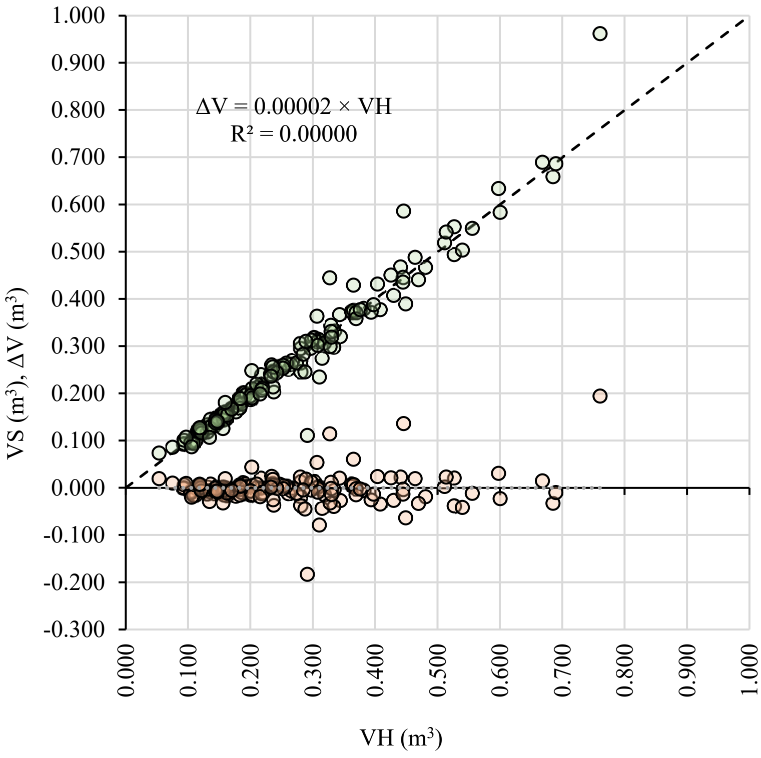

Figure S9. Trend of VS (green dots) and of the differences between the estimates (ΔV, brown dots) as a function of VH, plotted against the identity (1:1) line shown as the black dashed line. Legend: VH—volume estimated by Huber's formula, VS—volume estimated by Smalian's formula, ΔV—signed difference between VH and VS. Note: The sample contained 155 logs.

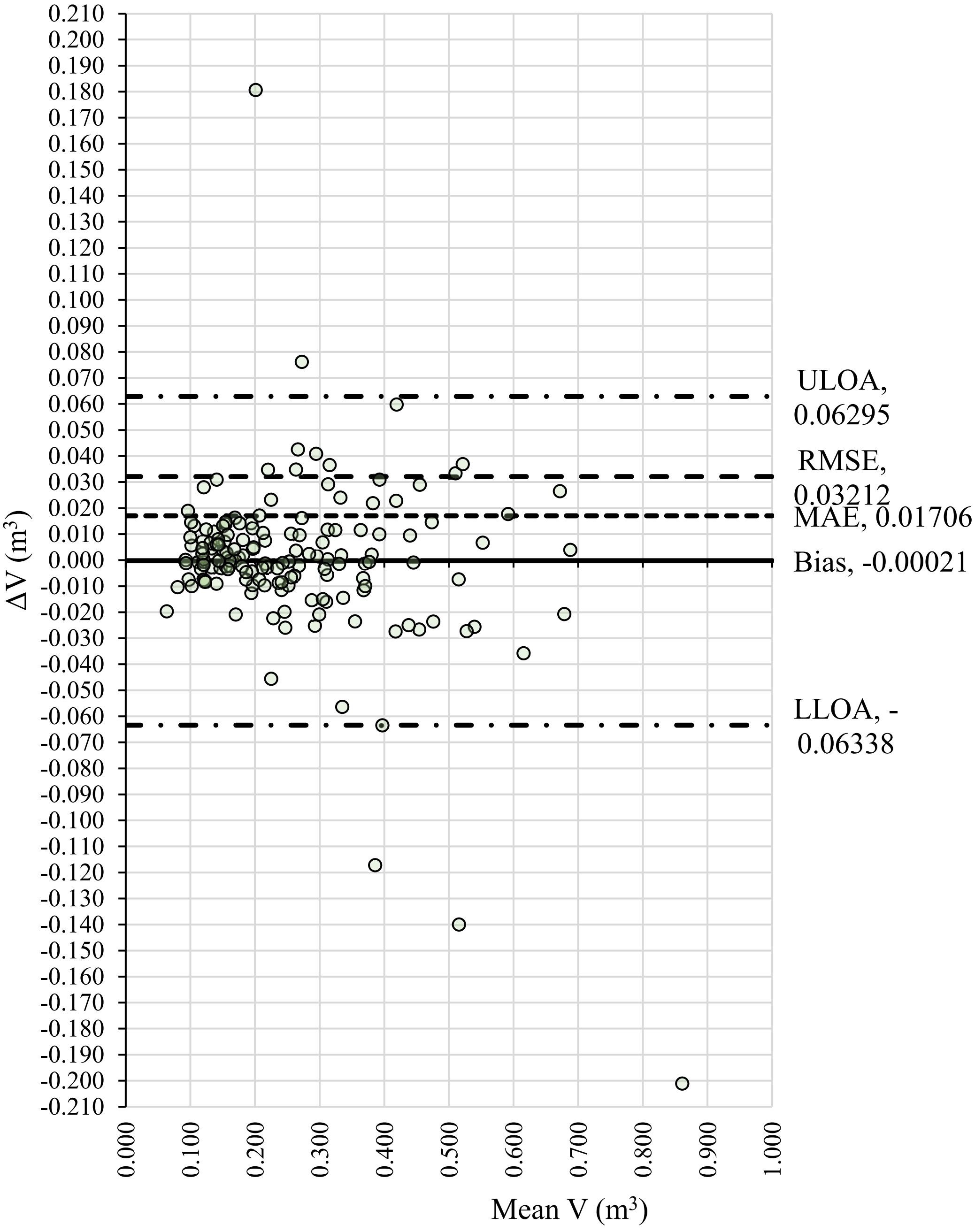

Figure S10. Bland–Altman plot showing the agreement of estimates between VH and VS. Legend: ΔV—signed differences between volume estimates, Mean V—average value of volume estimates, ULOA—upper limit of agreement, RMSE—root mean squared error, MAE—mean absolute error, Bias—bias, LLOA—lower limit of agreement. Note: There was good agreement when considering two standard deviations to compute the limits of agreement; the Bias was close to zero, meaning that there was low systematic bias. However, the mean absolute error was close to 0.02, and some observations large in magnitude led to an RMSE value of 0.03.

4. Internal consistency of the algorithm's estimates

Table S3. Results of heteroscedasticity tests.

Compared variables	Test	LM Stat	Degrees of freedom	p-value	Outcome
VAPP1_S1R1<br>VAPP2_S1R1	Breusch–Pagan	15.92	1	0.00007	heteroscedastic
VAPP1_S1R1<br>VAPP2_S1R1	White	16.86	2	0.00022	heteroscedastic
VAPP1_S1R2<br>VAPP2_S1R2	Breusch–Pagan	23.54	1	0.00000	heteroscedastic
VAPP1_S1R2<br>VAPP2_S1R2	White	23.54	2	0.00001	heteroscedastic
VAPP1_S2R1<br>VAPP2_S2R1	Breusch–Pagan	27.66	1	0.00000	heteroscedastic
VAPP1_S2R1<br>VAPP2_S2R1	White	27.66	2	0.00000	heteroscedastic
VAPP1_S2R2<br>VAPP2_S2R2	Breusch–Pagan	1.36	1	0.24399	homoscedastic
VAPP1_S2R2<br>VAPP2_S2R2	White	2.81	2	0.24507	homoscedastic

Note: The tests run were the Breusch–Pagan and White tests. Breusch–Pagan test is suited for detecting linear heteroscedasticity, whereas White's test can detect other forms of heteroscedasticity.

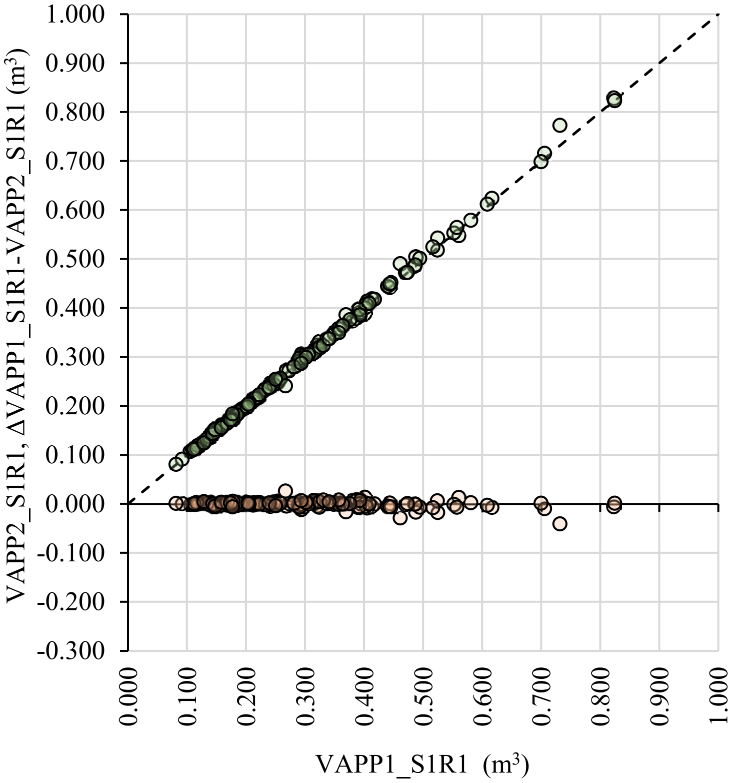

Figure S11. Scatterplot of VAPP1_S1R1 against VAPP2_S1R1. Legend: Green dots stand for the paired values of VAPP1_S1R1 and VAPP2_S1R1 and are plotted against the identity (1:1) line showed as black dashed line; brown dots stand for the signed difference between VAPP1_S1R1 and VAPP2_S1R1. Note: The sample contained 155 observations.

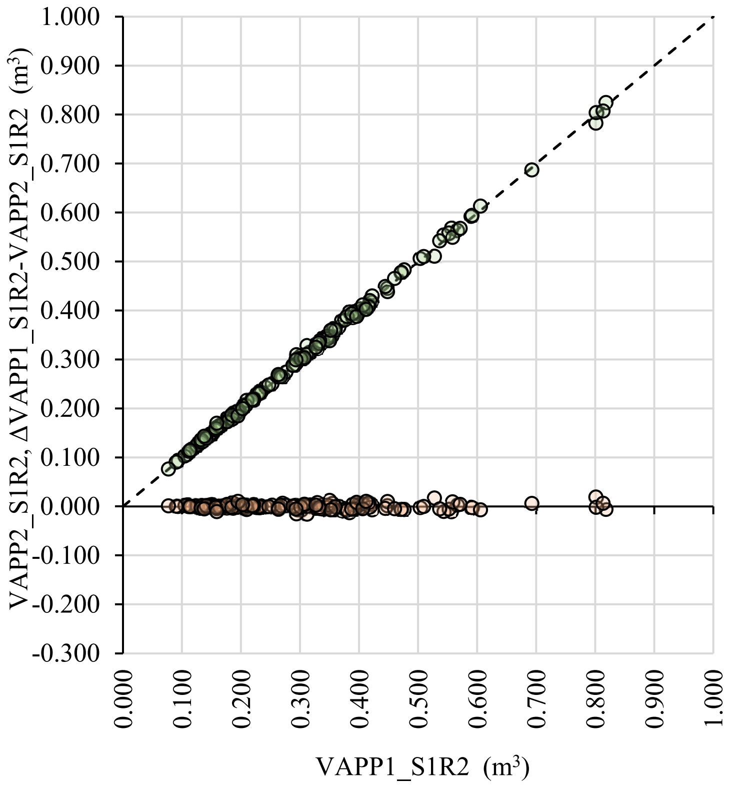

Figure S12. Scatterplot of VAPP1_S1R2 against VAPP2_S1R2. Legend: Green dots stand for the paired values of VAPP1_S1R2 and VAPP2_S1R2 and are plotted against the identity (1:1) line showed as black dashed line; brown dots stand for the signed difference between VAPP1_S1R2 and VAPP2_S1R2. Note: The sample contained 155 observations.

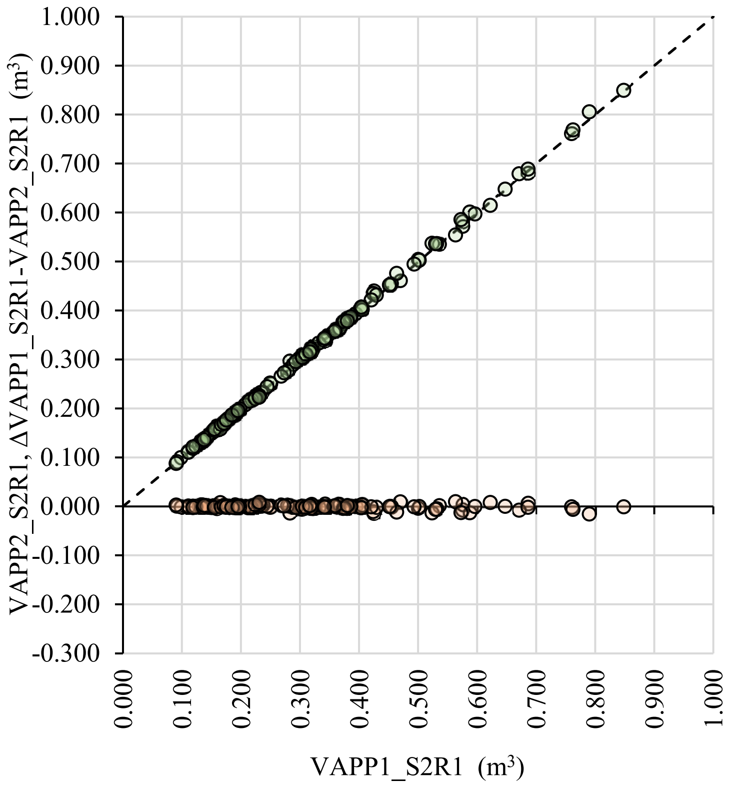

Figure S13. Scatterplot of VAPP1_S2R1 against VAPP2_S2R1. Legend: Green dots stand for the paired values of VAPP1_S2R1 and VAPP2_S2R1 and are plotted against the identity (1:1) line showed as black dashed line; brown dots stand for the signed differences between VAPP1_S2R1 and VAPP2_S2R1. Note: The sample contained 155 observations.

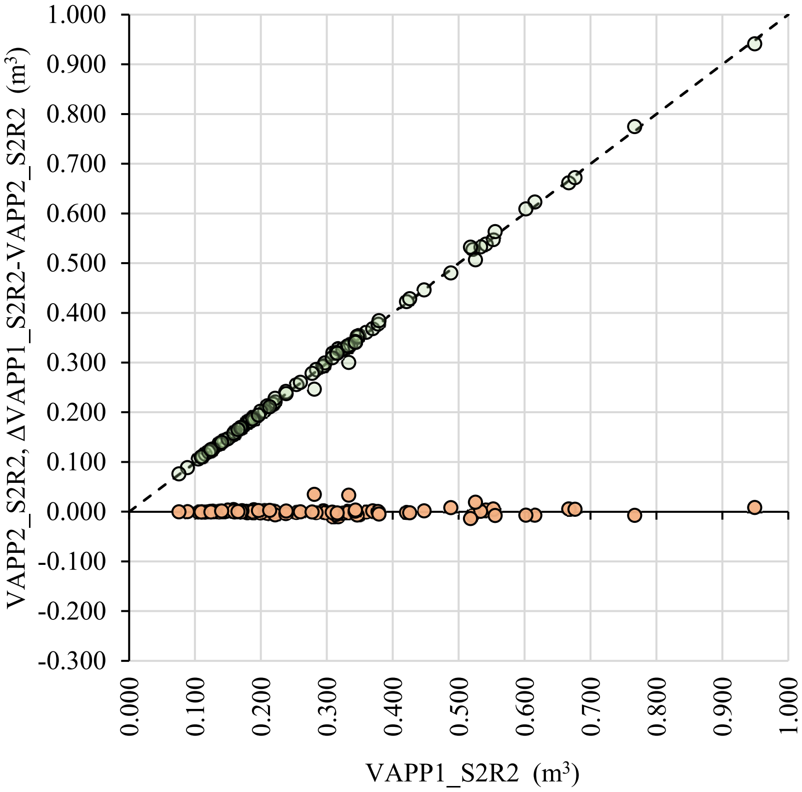

Figure S14. Scatterplot of VAPP1_S2R2 against VAPP2_S2R2. Legend: Green dots stand for the paired values of VAPP1_S2R2 and VAPP2_S2R2 and are plotted against the identity (1:1) line showed as black dashed line; brown dots stand for the signed differences between VAPP1_S2R2 and VAPP2_S2R2. Note: The sample contained 86 observations.

5. Intra-rater consistency

Table S4. Results of heteroscedasticity tests.

Compared variables	Test	LM Stat	Degrees of freedom	p-value	Outcome
VAPP1_S1R1<br>VAPP1_S1R2	Breusch–Pagan	11.83	1	0.00058	heteroscedastic
VAPP1_S1R1<br>VAPP1_S1R2	White	12.49	2	0.00194	heteroscedastic
VAPP2_S1R1<br>VAPP2_S1R2	Breusch–Pagan	11.75	1	0.00061	heteroscedastic
VAPP2_S1R1<br>VAPP2_S1R2	White	13.40	2	0.00123	heteroscedastic
VAPP1_S2R1<br>VAPP1_S2R2	Breusch–Pagan	2.72	1	0.09926	homoscedastic
VAPP1_S2R1<br>VAPP1_S2R2	White	3.81	2	0.14851	homoscedastic
VAPP2_S2R1<br>VAPP2_S2R2	Breusch–Pagan	2.85	1	0.09136	homoscedastic
VAPP2_S2R1<br>VAPP2_S2R2	White	2.88	2	0.13282	homoscedastic

Note: The tests run were the Breusch–Pagan and White tests. Breusch–Pagan test is suited for detecting linear heteroscedasticity, whereas White's test can detect other forms of heteroscedasticity.

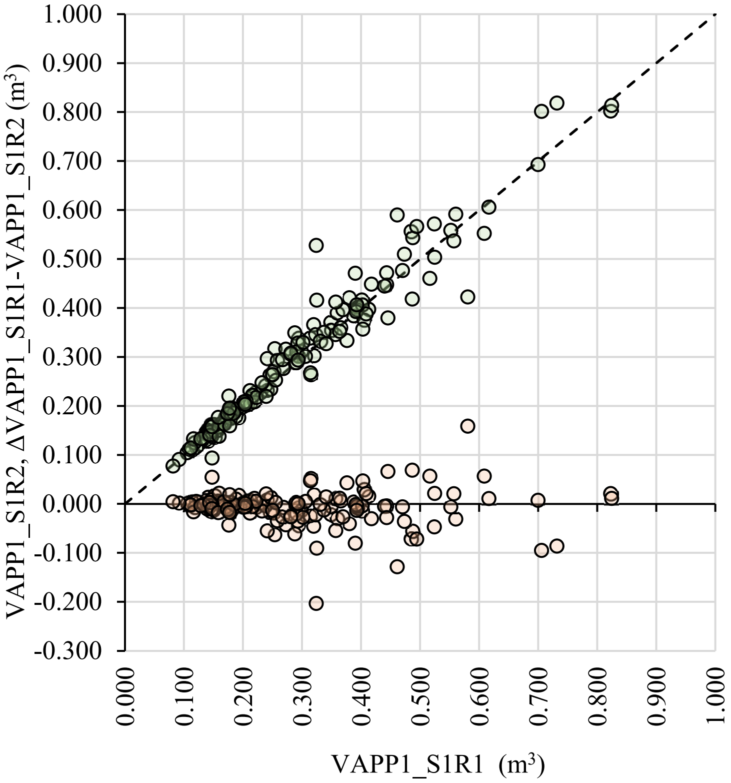

Figure S15. Scatterplot of VAPP1_S1R1 against VAPP1_S1R2. Legend: Green dots stand for the paired values of VAPP1_S1R1 and VAPP1_S1R2 and are plotted against the identity (1:1) line showed as black dashed line; brown dots stand for the signed difference between VAPP1_S1R1 and VAPP1_S1R2. Note: The sample contained 155 observations.

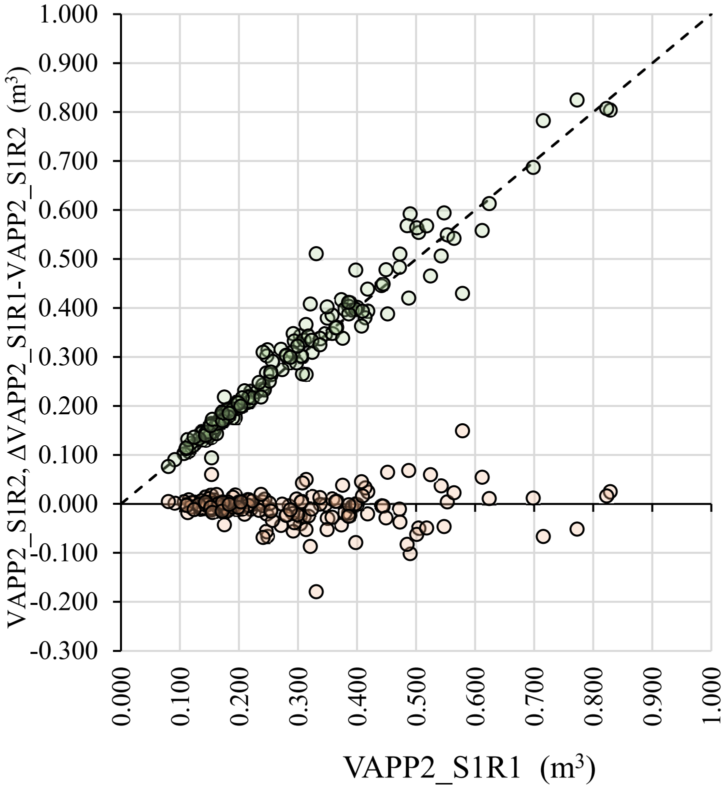

Figure S16. Scatterplot of VAPP2_S1R1 against VAPP2_S1R2. Legend: Green dots stand for the paired values of VAPP2_S1R1 and VAPP2_S1R2 and are plotted against the identity (1:1) line showed as black dashed line; brown dots stand for the signed difference between VAPP2_S1R1 and VAPP2_S1R2. Note: The sample contained 155 observations.

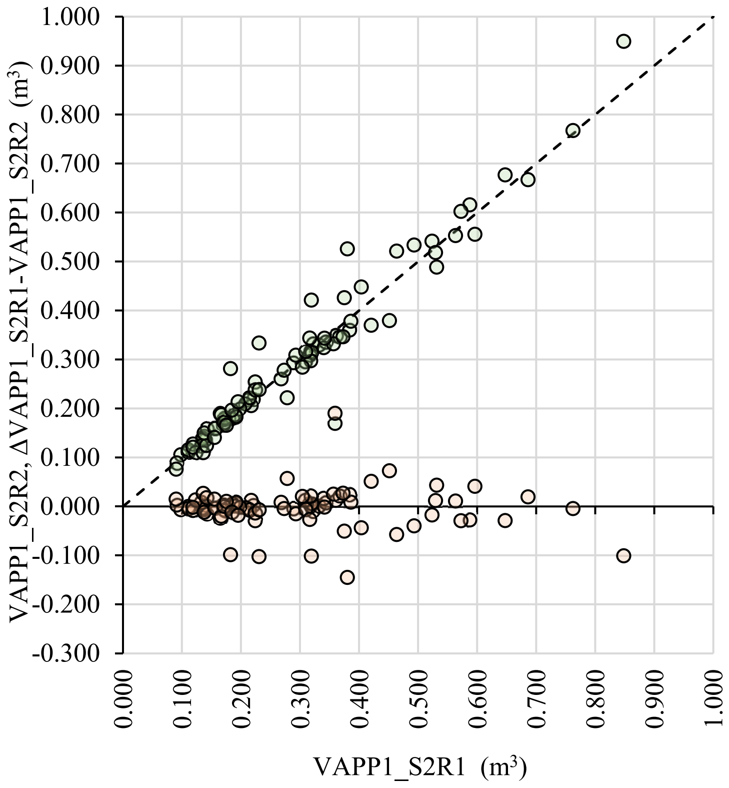

Figure S17. Scatterplot of VAPP1_S2R1 against VAPP1_S2R2. Legend: Green dots stand for the paired values of VAPP1_S2R1 and VAPP1_S2R2 and are plotted against the identity (1:1) line showed as black dashed line; brown dots stand for the signed difference between VAPP1_S2R1 and VAPP1_S2R2. Note: The sample contained 89 observations.

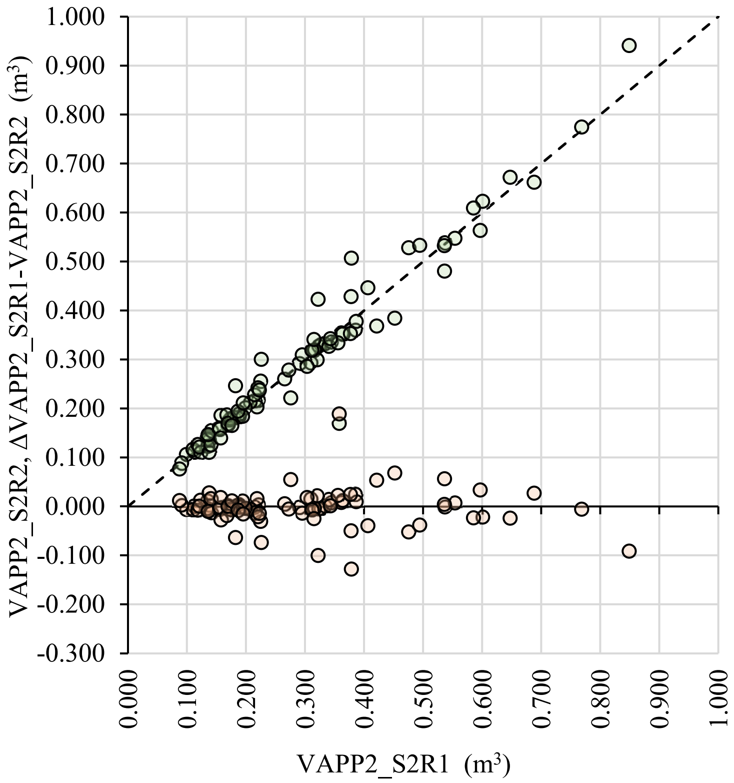

Figure S18. Scatterplot of VAPP2_S2R1 against VAPP2_S2R2. Legend: Green dots stand for the paired values of VAPP2_S2R1 and VAPP2_S2R2 and are plotted against the identity (1:1) line showed as black dashed line; brown dots stand for the signed difference between VAPP2_S2R1 and VAPP2_S2R2. Note: The sample contained 89 observations.

6. Inter-rater consistency

Table S5. Results of heteroscedasticity tests.

Compared variables	Test	LM Stat	Degrees of freedom	p-value	Outcome
VAPP1_S1R1 VAPP1_S2R1	Breusch–Pagan	18.68	1	0.00002	heteroscedastic
VAPP1_S1R1 VAPP1_S2R1	White	18.87	2	0.00008	heteroscedastic
VAPP2_S1R1 VAPP2_S2R1	Breusch–Pagan	22.81	1	0.00000	heteroscedastic
VAPP2_S1R1 VAPP2_S2R1	White	23.25	2	0.00001	heteroscedastic
VAPP1_S1R2 VAPP1_S2R2	Breusch–Pagan	10.97	1	0.00092	heteroscedastic
VAPP1_S1R2 VAPP1_S2R2	White	11.57	2	0.00308	heteroscedastic
VAPP2_S1R2 VAPP2_S2R2	Breusch–Pagan	10.37	1	0.00128	heteroscedastic
VAPP2_S1R2 VAPP2_S2R2	White	11.68	2	0.00294	heteroscedastic

Note: The tests run were the Breusch–Pagan and White tests. Breusch–Pagan test is suited for detecting linear heteroscedasticity, whereas White's test can detect other forms of heteroscedasticity.

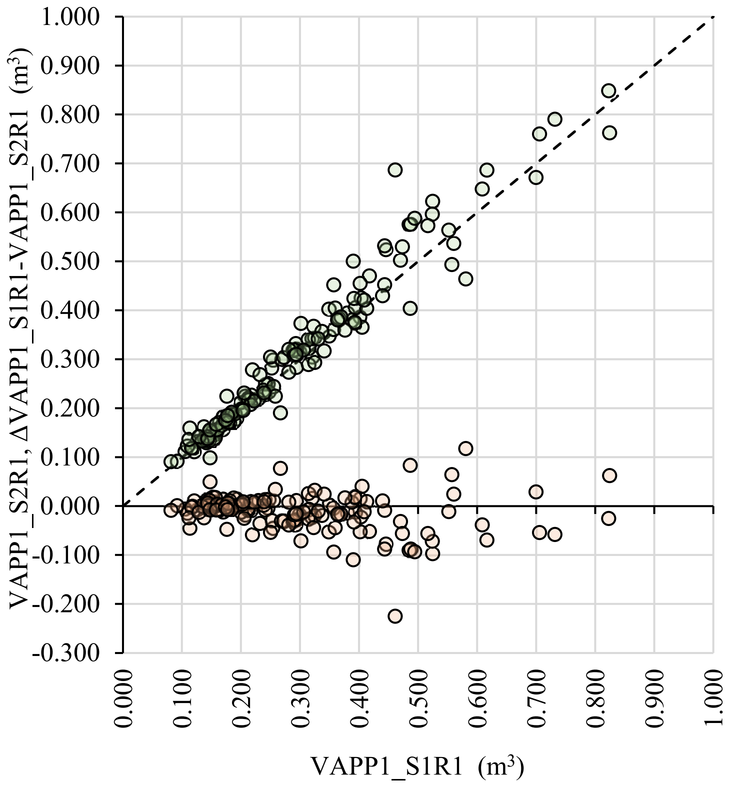

Figure S19. Scatterplot of VAPP1_S1R1 against VAPP1_S2R1. Legend: Green dots stand for the paired values of VAPP1_S1R1 and VAPP1_S2R1 and are plotted against the identity (1:1) line showed as black dashed line; brown dots stand for the signed difference between VAPP1_S1R1 and VAPP1_S2R1. Note: The sample contained 155 observations.

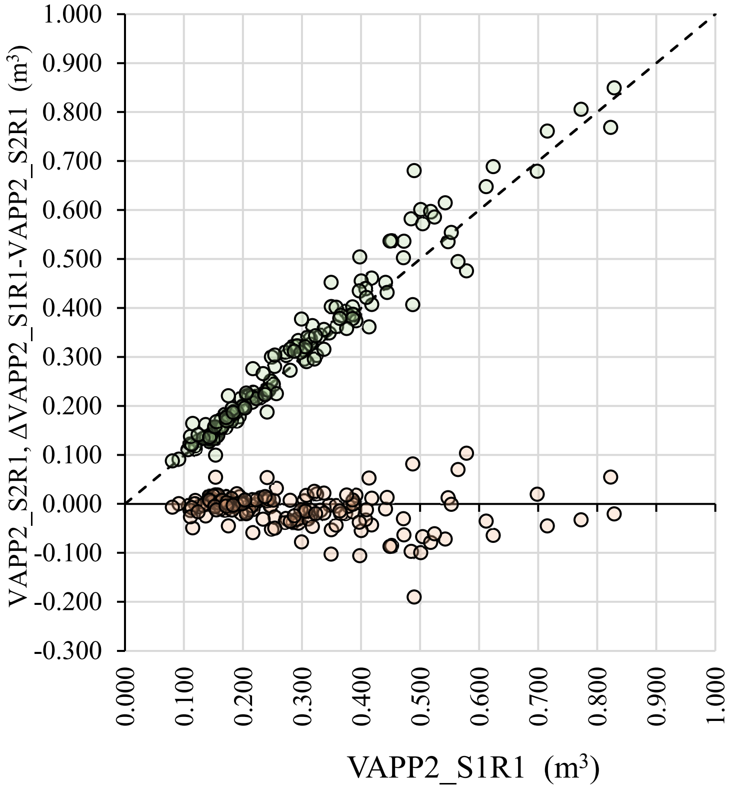

Figure S20. Scatterplot of VAPP2_S1R1 against VAPP2_S2R1. Legend: Green dots stand for the paired values of VAPP2_S1R1 and VAPP2_S2R1 and are plotted against the identity (1:1) line showed as black dashed line; brown dots stand for the signed difference between VAPP2_S1R1 and VAPP2_S2R1. Note: The sample contained 155 observations.

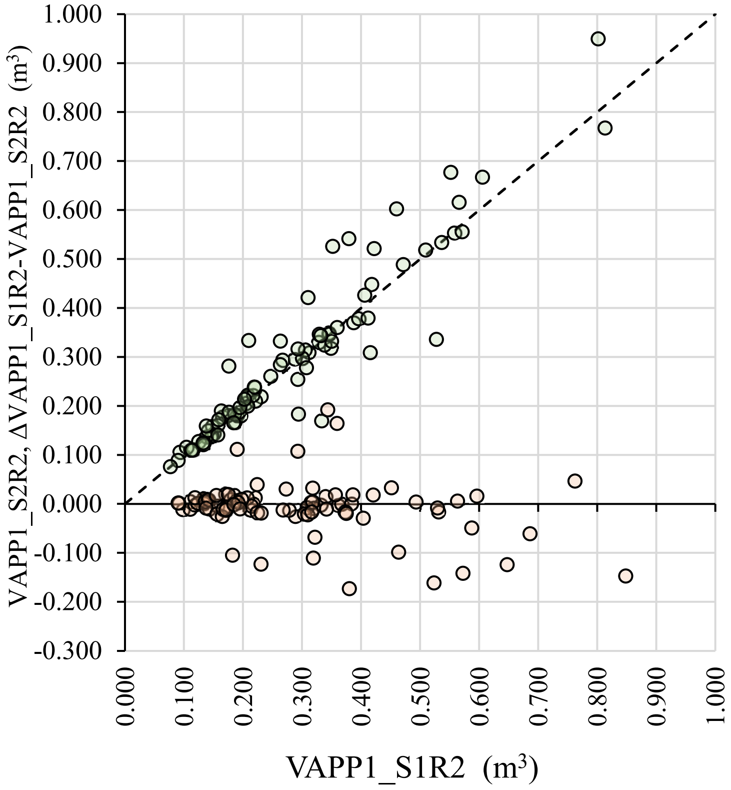

Figure S21. Scatterplot of VAPP1_S1R2 against VAPP1_S2R2. Legend: Green dots stand for the paired values of VAPP1_S1R2 and VAPP1_S2R2 and are plotted against the identity (1:1) line showed as black dashed line; brown dots stand for the signed difference between VAPP1_S1R2 and VAPP1_S2R2. Note: The sample contained 89 observations.

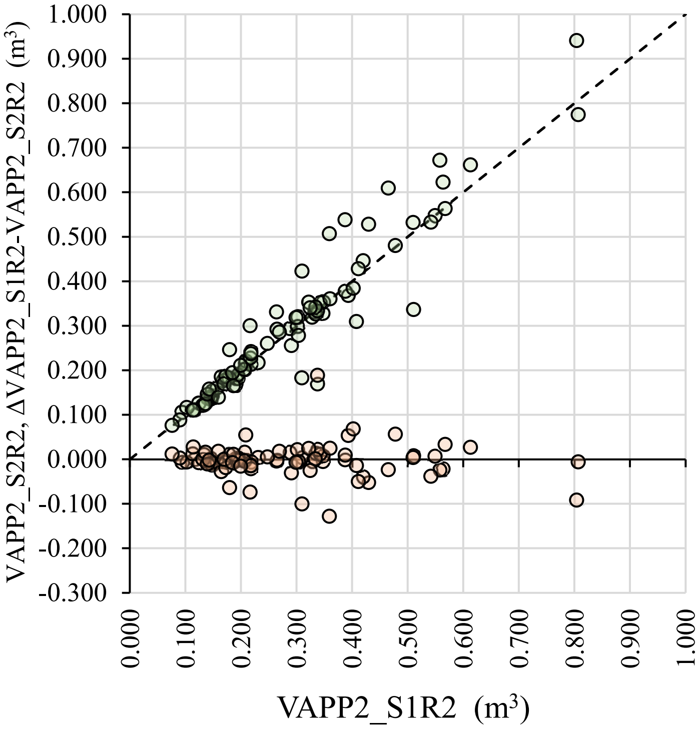

Figure S22. Scatterplot of VAPP2_S1R2 against VAPP2_S2R2. Legend: Green dots stand for the paired values of VAPP2_S1R2 and VAPP2_S2R2 and are plotted against the identity (1:1) line showed as black dashed line; brown dots stand for the signed difference between VAPP2_S1R2 and VAPP2_S2R2. Note: The sample contained 89 observations.

7. Agreement between digital and manual estimates

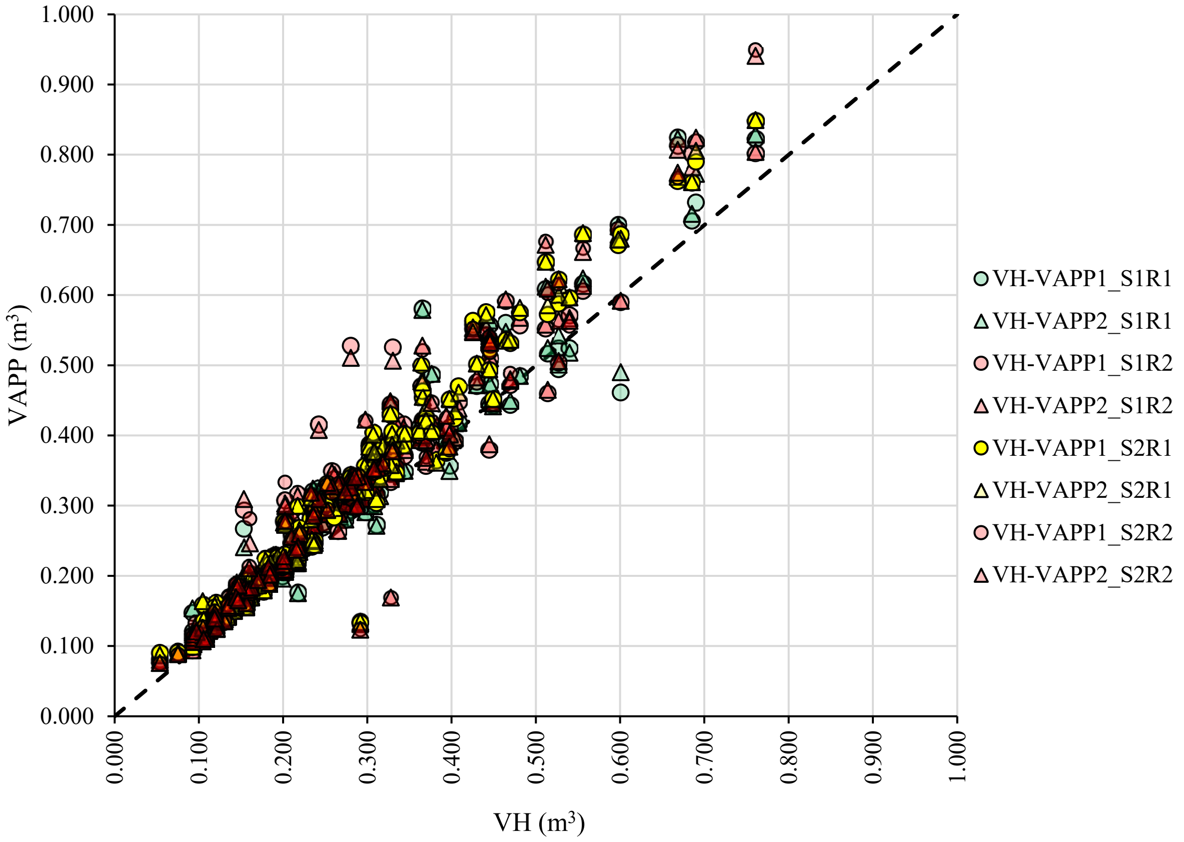

Figure S23. Scatterplot of VAPP variables against VH. Note: Shapes of various colors stand for the paired values of the eight datasets (see the figure legend) against the VH estimates and are plotted against the identity (1:1) line showed as black dashed line. Note: The sample contained 89 observations when using VAPP1_S2R2 and VAPP2_S3R2 datasets.

8. Time efficiency

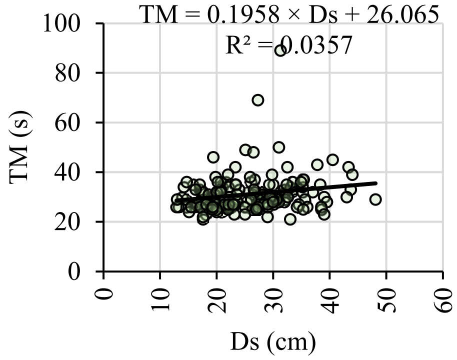

a

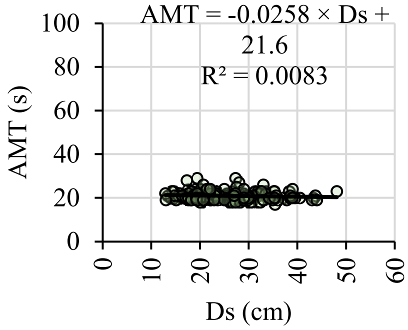

b

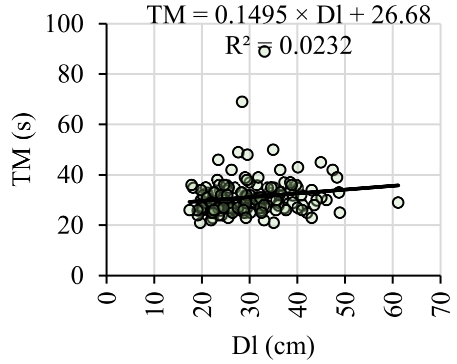

c

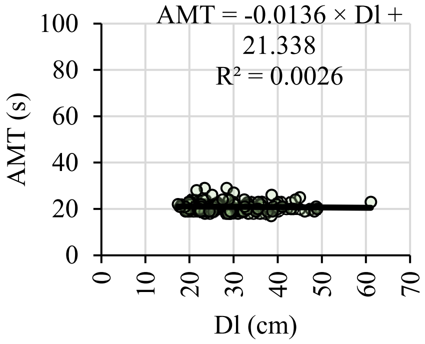

d

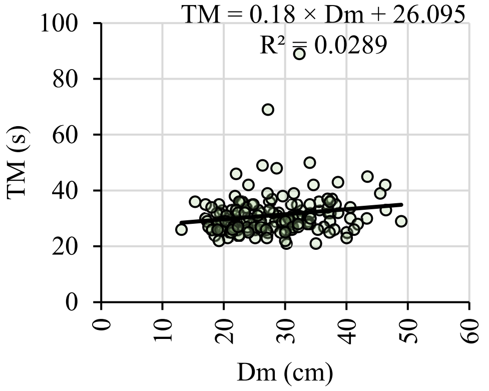

e

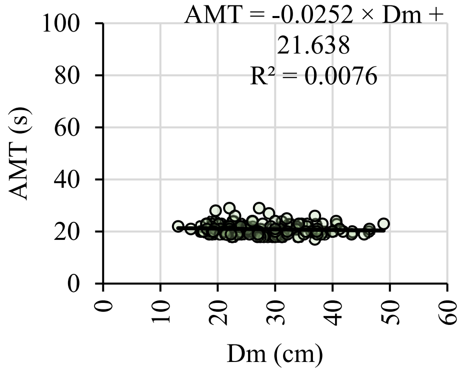

f

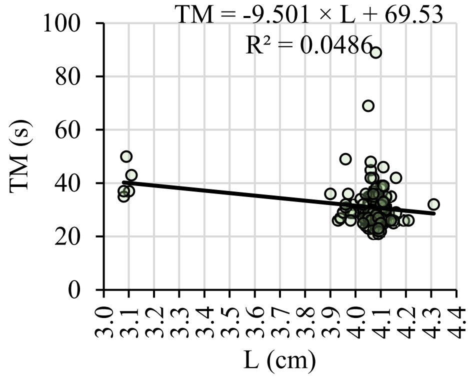

g

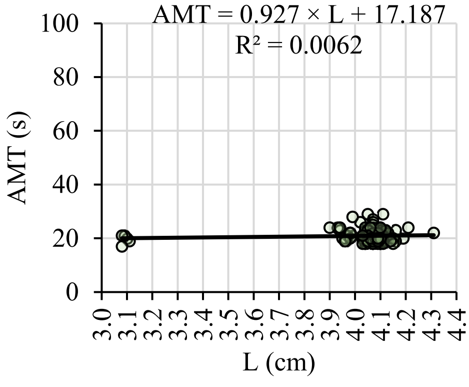

h

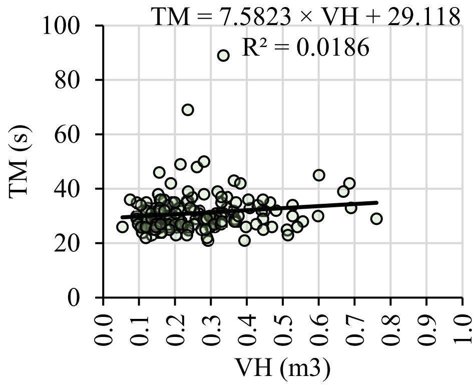

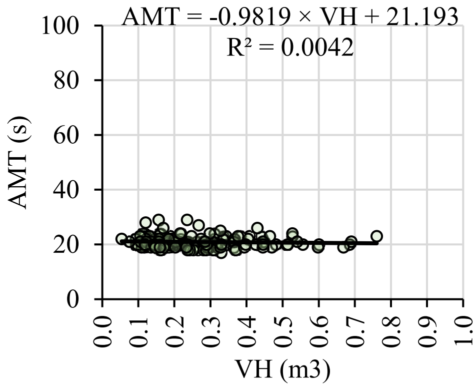

i j

Figure S24. Dependence of time consumption for manual (TM) and digital (AMT) measurements on variability of log biometrics collected manually. Legend: TM—cycle time for manual measurement, AMT—cycle time for digital measurement, Ds—diameter at the small end, Dl—diameter at the large end, Dm—diameter at the middle, L—log length, VH—volume estimated by Huber's formula.
